# Testicular Disorders in Equids: A Case Series

**DOI:** 10.3390/ani16152439

**Published:** 2026-08-06

**Authors:** Giuseppe Catone, Valentina Palmieri, Giada Giambrone, Gabriele Marino, Gian Enrico Magi, Francesca Mariotti, Cecilia Vullo, Michela Ciccarelli

**Affiliations:** 1 Department of Veterinary Sciences, University of Messina, 98168 Messina, Italy; gcatone@unime.it (G.C.); valentina.palmieri1@studenti.unime.it (V.P.);; 2 School of Biosciences and Veterinary Medicine, University of Camerino, 62024 Macerata, Italy; 3 Department of Chemical, Biological, Pharmaceutical and Environmental Sciences, University of Messina, 98166 Messina, Italy; 4 College of Veterinary Medicine, Washington State University, Pullman, WA 99164, USA; 5 School of Molecular Bioscience, Washington State University, Pullman, WA 99164, USA

**Keywords:** horse, testis, DSDs, spermatic cord torsion, orchitis, neoplasia, ultrasonography, histopathology

## Abstract

Equine testicular disorders represent a diverse group of conditions that impact the longevity of a stallion’s breeding career. Stud farm veterinarians should be familiar with the variety of clinical presentations and diagnostic options for these conditions. This retrospective case series reports 57 cases of testicular abnormalities in stallions and donkeys presented to the clinic for further diagnostics and treatment. These disorders included neoplasia (three unilateral and one bilateral seminoma; two Sertoli cell tumors; two Leydig cell tumors; and one malignant mixed sex cord–stromal tumor), six cases of testicular hypoplasia and disorders of sexual development (DSDs), twenty-four cases of cryptorchidism with compensatory hypertrophy/hyperplasia, and two cases of monorchidism. Among the monorchid horses, one of these involved an abdominal retained testicle with a concurrent Leydig cell tumor, whereas the second presented a normally scrotal testis. Additional diagnoses consisted of testicular degeneration, including suspected anabolic steroid-induced hypogonadism, spermatic cord torsion, hydroceles, periorchitis, scrotal sarcoid associated with severe testicular degeneration, parasitic orchitis and periorchitis associated with *Strongylus vulgaris* and *Setaria equina*, one infectious epididymitis with orchitis in a donkey, and one case of ectopic adrenal cortical tissue within the testicular parenchyma. Rarely observed disorders included cystic ectasia of the rete testis in a cryptorchid testis and ischemic necrosis following failed laparoscopic castration without orchidectomy. This case series reviews the pathophysiology, clinical presentation, diagnostic techniques, and outcomes of various testicular diseases, providing a guide to facilitate case management in private practice. Additionally, it offers histopathologic descriptions of most cases, which are often missing from routine diagnostics but are crucial for definitive diagnosis. These findings demonstrate that horse testicular diseases encompass a wide range of neoplastic, developmental, degenerative, infectious, and parasitic conditions that can coexist and often go unnoticed until they progress.

## Simple Summary

Testicular diseases in horses encompass a broad spectrum of developmental, degenerative, inflammatory, parasitic, and neoplastic conditions that can compromise fertility and breeding performance. This study presents one of the largest single-institution case series describing testicular disorders in equids, collected over a 26-year period at two Veterinary Teaching Hospitals. The cases include congenital abnormalities, disorders of sexual development, cryptorchid testes, testicular tumors, spermatic cord torsion, hydrocele, inflammatory and parasitic diseases, and several uncommon lesions rarely reported in the literature, including a rare case of bacterial epididymitis with orchitis in a donkey. Each case was investigated using a combination of clinical examination, ultrasonography, surgical exploration, gross pathology, and cytogenetic analysis when indicated, and, when available, histopathology, yielding definitive diagnoses and detailed lesion characterization. By integrating these complementary diagnostic approaches, this work offers a comprehensive overview of the spectrum of testicular diseases affecting equids and provides practical information to support veterinarians in their diagnosis and clinical management. The findings also emphasize the importance of pathological examination of excised testes, as clinically unsuspected lesions may otherwise remain undetected.

## 1. Introduction

Reproductive disorders are common in equids and account for a substantial proportion of the clinical caseload seen by veterinary practitioners. Among these, testicular disorders constitute a heterogeneous group of conditions that may impair fertility, compromise breeding soundness, and require medical or surgical intervention [1]. Although numerous case reports on individual equine testicular disorders have been published, comprehensive retrospective studies evaluating the spectrum of clinical presentations, diagnostic imaging, surgical findings, and histopathology across multiple cases remain limited.

Most of these pathologies are acquired and present with scrotal enlargement, including orchitis, epididymitis, some testicular neoplasia, hydrocele, spermatic cord torsion, inguinal/scrotal herniation, and trauma. Some conditions may reduce testicular size, as in testicular degeneration [2,3,4]. Additionally, several congenital abnormalities occur in equids, including disorders of sex development, cystic ectasia of the rete testis, and ectopic adrenal tissue. In stallions, testicular degeneration is a common cause of acquired subfertility and infertility [2]. Usually, this condition is suspected after semen collection, when sperm quality is poor, and various morphological abnormalities and round cells are found, indicating damage to the seminiferous tubules and premature shedding of the seminiferous epithelium, respectively. Ultrasonography reveals a heterogeneous parenchyma that may display hyperechoic trabeculae extending from the rete testis, which is consistent with fibrosis. The potential for reversing testicular degeneration depends on the root cause, severity, and early intervention. In such cases, animals are typically reevaluated after about 60 days to allow for a new spermatogenic cycle.

Disorders of sex development (DSDs) comprise a heterogeneous group of congenital conditions characterized by discordance among chromosomal, gonadal, phenotypic, and hormonal sex [5,6]. Equine DSDs are recognized as one of the most important non-infectious causes of infertility, subfertility, and congenital reproductive abnormalities, accounting for approximately 30% of horses presented with abnormal behavior or phenotypic characteristics of the external genitalia. These disorders are frequently associated with sex chromosome abnormalities [7,8,9].

DSDs have recently been reclassified based on karyotype and SRY status, including monosomy X (63, XO) and sex chromosome mosaicism (64, XX SRY-negative; 64, XY SRY-positive; and 64, XY SRY-negative) [10].

Cryptorchidism is the most common disorder of sex development in equids, with a prevalence estimated to be between 2% and 8% [11]. Affected males are usually presented due to stallion-like behavior, even if purchased as geldings. Surgical removal is generally recommended because retained testes have an increased risk of spermatic cord torsion, either primary or secondary to neoplasia [12,13], orchitis associated with *Strongylus* spp. infection [13,14], neoplasia, and cystic dilation of the rete testis.

Cystic dilation of the rete testis and ectopic adrenal tissue [6,15,16,17] are uncommon developmental anomalies that have been reported only occasionally in horses and other species. These are incidental findings during breeding soundness exams and post-castration.

Scrotal enlargement is the most common clinical presentation for equine cases. Although often the result of direct trauma to the scrotum, regional herniation and spermatic cord torsion are relatively common. Less common causes include infections, hydrocele, hematocele or pyocele, and neoplasia [18].

Spermatic cord torsion is an uncommon condition but occurs more frequently in stallions than in other domestic species, likely because the longer caudal ligament of the epididymis and proper ligament of the testis allow greater testicular mobility [12,16,19,20]. Affected stallions often have a history of intermittent, mild episodes of colic associated with partial torsion that spontaneously resolves, followed by the sudden onset of severe abdominal pain when torsion exceeds approximately 270°. Although acute pain is the most common clinical presentation, some stallions may instead present with painless scrotal enlargement if the initial painful episode goes unrecognized. In these cases, prolonged vascular and neural compromise results in loss of sensation and the development of hydrocele, hematocele, severe testicular degeneration, and coagulative hemorrhagic necrosis.

Enlargement of an otherwise normal testis is rarely considered a primary disorder. In most cases, unilateral testicular enlargement represents secondary or compensatory hypertrophy associated with an underlying pathological condition affecting the contralateral testis, such as orchitis, neoplasia, hypoplasia, cryptorchidism, atrophy, or previous hemicastration [6]. In contrast, reduction in testicular size is more commonly associated with testicular atrophy or hypotrophy secondary to degeneration, endocrine dysfunction, aging or exposure to toxic substances.

Hydrocele is characterized by an abnormal accumulation of serous fluid between the parietal and visceral layers of the tunica vaginalis [18]. It has also been reported more frequently in hot and humid environments, where alterations in spermatic cord hemodynamics may promote leakage of peritoneal fluid into the vaginal cavity [21]. Clinically, affected stallions typically present with painless scrotal enlargement, and palpation reveals fluctuant fluid surrounding a small- to normal-sized testis [18]. Ultrasonography is the imaging modality of choice, demonstrating anechoic fluid within the vaginal cavity surrounding the testis and epididymis, while centesis generally yields a clear amber serous fluid [18]. In any case, disruption of temperature regulation can cause testicular degeneration and infertility; therefore, typical treatments include cold hosing of the scrotum, controlled exercise, and anti-inflammatory drugs.

Orchitis and epididymitis are uncommon inflammatory disorders of the equine reproductive tract [22,23,24]. Bacterial orchitis has been associated with pathogens including *Streptococcus equi* subsp. *zooepidemicus*, *Actinobacillus equuli*, *Burkholderia mallei*, *Salmonella abortus equi*, *Escherichia coli*, and *Corynebacterium pseudotuberculosis*, whereas bacterial epididymitis is most commonly caused by *Streptococcus equi* subsp. *zooepidemicus* and *Proteus mirabilis* [6,22,23,24].

Testicular tumors are uncommon in horses, accounting for approximately 0.04–0.9% of all equine neoplasms [25]. However, their true incidence is likely underestimated because most stallions are castrated at a young age, and excised testicular tissue is infrequently submitted for histopathological evaluation [26].

This retrospective case series describes the clinical presentation, diagnostic findings, surgical management, and gross pathological and histopathological features of testicular disorders diagnosed in equids over a 26-year period at the Veterinary Teaching Hospitals of the Universities of Camerino and Messina. By compiling a large series of uncommon testicular disorders, this study aims to improve recognition of their clinical presentation, facilitate differential diagnosis, and provide practical guidance for the diagnostic evaluation, treatment, and management of affected horses.

## 2. Case Selection

A retrospective review of medical records was conducted on equids diagnosed with testicular disorders and treated at the Veterinary Teaching Hospitals of the University of Camerino and the University of Messina between January 2000 and June 2026.

Case management was not influenced by the study; therefore, ethical approval was not required. Owners signed an informed consent form authorizing hospitalization and treatment, and additional consent was obtained for the use of biological samples for research purposes.

The survey was conducted using the clinical archives of equids referred to the Veterinary Teaching Hospitals. Only cases with a definitive diagnosis of testicular disorders, established on the basis of clinical, ultrasonographic, surgical, gross pathological, and, when available, histopathological findings, were included in the study. All testicular disorders included in the present case series were confirmed following orchiectomy.

Ultrasonographic examination of the testes was performed using either a 3.5 MHz convex transducer for inguinal examinations or a 7.5 MHz linear transducer for transrectal and scrotal examinations. All examinations were carried out using SonoSite Titan, MicroMaxx, and M-Turbo ultrasound systems (FUJIFILM Sonosite Inc., Bothell, WA, USA).

The surgical approach was selected according to the nature and location of the lesion. Minimally invasive laparoscopic procedures were primarily used to treat abdominal cryptorchid testes. Horses with inguinal cryptorchidism were generally managed using a laparoscopic-assisted inguinal approach, consisting of standing laparoscopy for identification and transection of the spermatic cord, followed by removal of the retained testis through an inguinal incision under general anesthesia. Normally descended testes were treated by unilateral or bilateral orchiectomy using standard open or closed castration techniques, performed either under general anesthesia in dorsal recumbency or in the standing sedated horse depending on the clinical indication and surgeon’s preference.

Excised tissues were submitted for gross pathological examination and, whenever available, histopathological evaluation.

For histopathological evaluation, the excised samples were fixed in 10% neutral buffered formalin for 48 h. Following fixation, the tissues were dehydrated through graded ethanol solutions (70%, 80%, and 95%) for 2–3 h each, cleared in xylene for 4 h, and embedded in paraffin wax for 4 h. Sections 5 μm thick were cut using a microtome, mounted on glass slides, stained with hematoxylin and eosin (H&E), and examined using a light microscope.

In cases where testicular parasites were identified, they were gently isolated with tweezers under a stereomicroscope, rinsed twice in sterile saline, and stored in 70% ethanol for morphological identification. Before identification, the parasites were cleared in glycerin for 24 h, mounted on temporary slides in glycerin, and examined under a light microscope. Species identification was performed according to morphometric keys [27,28,29].

## 3. Results

### 3.1. Congenital Disorders

#### 3.1.1. Disorders of Sexual Development (DSDs)

Six horses were diagnosed with disorders of sexual development (DSDs), representing a heterogeneous group of congenital abnormalities characterized by abnormal external genitalia, testicular hypoplasia, and variable cytogenetic findings (Table 1). All horses underwent ultrasonographic examination followed by surgical exploration. Depending on gonadal location, treatment consisted of standing laparoscopic cryptorchidectomy, closed inguinal castration under general anesthesia, or combined laparoscopic cryptorchidectomy for the abdominal gonad and standing laparoscopy to identify, desensitize, and transect the spermatic cord intra-abdominally, followed by gonadectomy through an inguinal incision under general anesthesia. Histopathological examination confirmed severe bilateral testicular hypoplasia in five of the six horses. Cytogenetic analysis was available in four cases and demonstrated considerable variability, including a normal male karyotype (64, XY SRY-positive), chromosomal mosaicism (64, XX/64, XY/63, XO SRY-positive), and a 64, XX SRY-negative karyotype.

**Figure 1:**
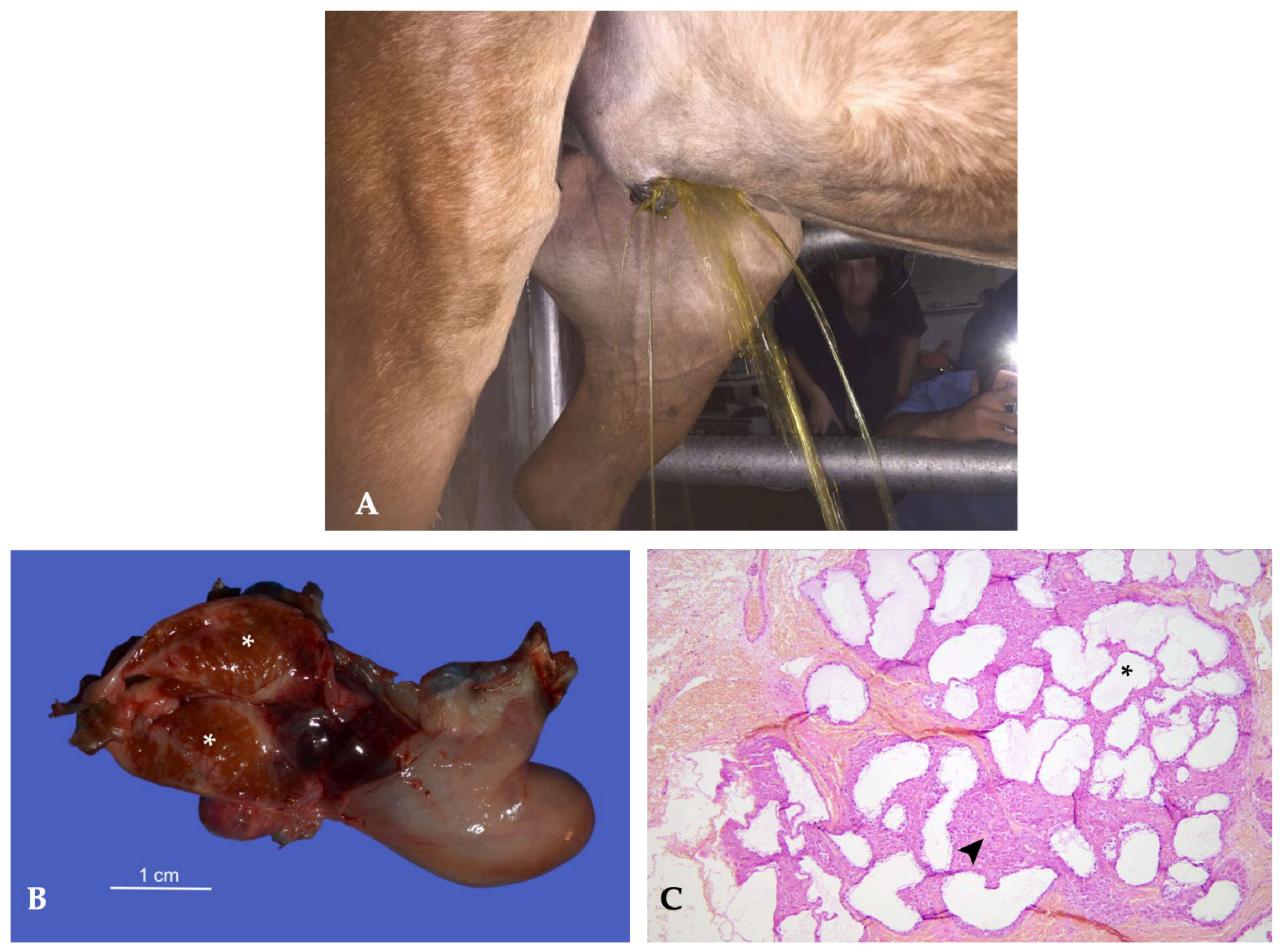
**Case 1.** Five-year-old Arabian stallion with DSDs (64, XY SRY-positive). (**A**) External genitalia during urination (shower-like) showing a reduced prepuce, micropenis, and complete absence of the scrotal sac. (**B**) Gross appearance of one hypoplastic abdominal gonad following laparoscopic cryptorchidectomy. Asterisk indicates the rudimentary testicular tissue. (**C**) Histological section of testis with diffuse and severe atrophy of seminiferous tubules. The tubular epithelium is flattened and completely devoid of germinal activity (asterisk), whereas the peritubular interstitium is expanded by aggregates of interstitial cells (arrowhead) (H&E, 10×).

Case 1 was a 5-year-old Arabian horse referred for suspected bilateral cryptorchidism and complete absence of libido. Physical examination revealed an underdevelopment of the prepuce and penis (Figure 1A), complete absence of the scrotal sac, and no palpable testes. Transrectal ultrasonography identified tubular structures dorsal to the urinary bladder, presumed to represent the ampullae of the ductus deferens, whereas no gonads were visualized within the abdomen or inguinal canals. Standing laparoscopy identified two small abdominal gonads (approximately 1.5 × 1 cm), which were removed laparoscopically (Figure 1B). Histopathological examination demonstrated severe diffuse testicular hypoplasia with marked degenerative changes and extensive fibrovascular replacement of the testicular parenchyma (Figure 1C). Cytogenetic analysis demonstrated a normal male karyotype (64, XY) with a positive SRY gene.

Cases 2–5 shared a remarkably similar clinical phenotype characterized by increased anogenital distance, abnormal external genitalia with a small penis located in the perineal region, complete absence of the scrotum, bilateral testicular hypoplasia, and persistent stallion-like behavior. Histopathological examination consistently demonstrated diffuse testicular hypoplasia. The principal differences among these horses were related to the gonadal location, cytogenetic findings, and associated congenital abnormalities.

**Figure 2:**
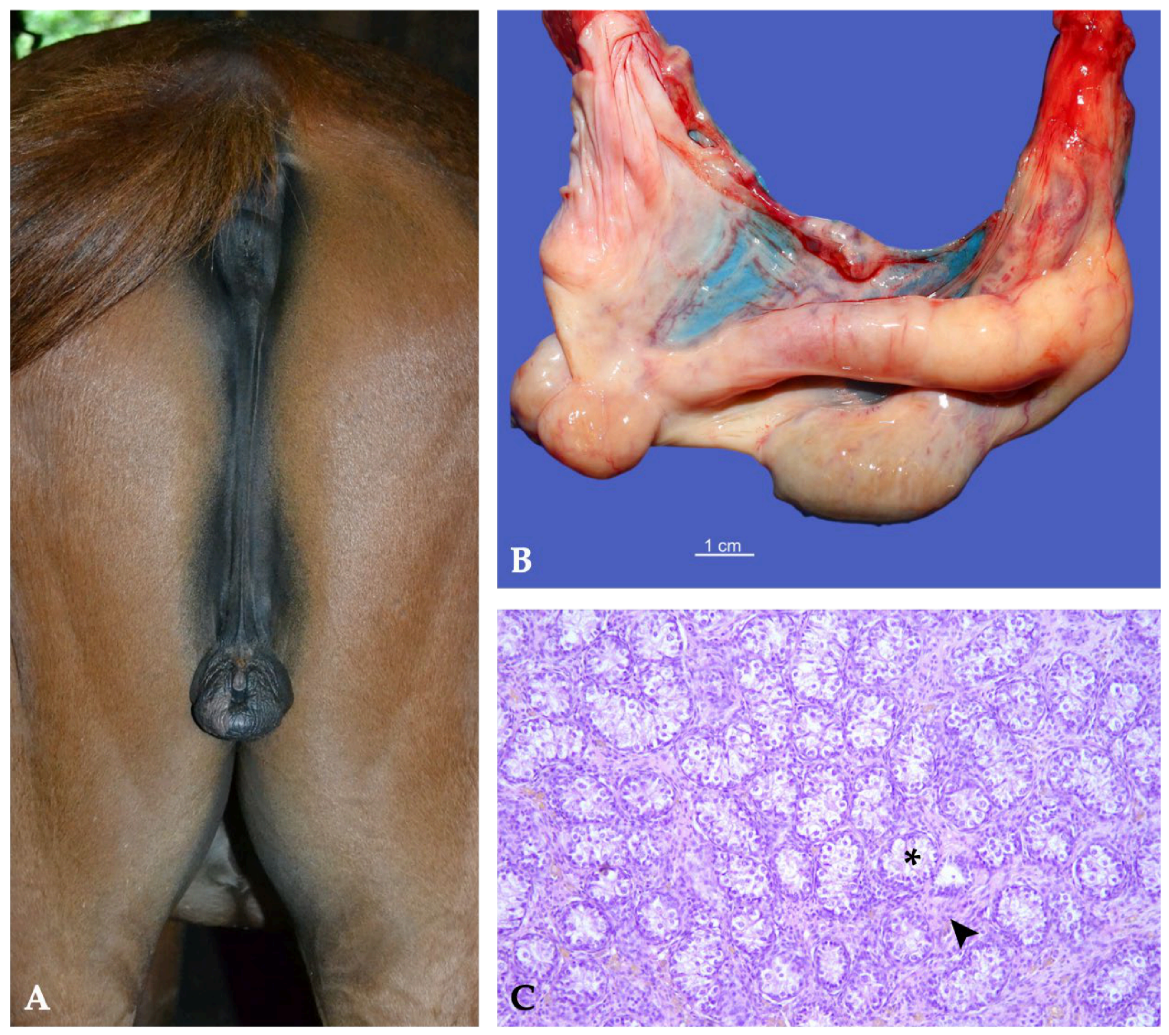
**Case 2.** Fifteen-month-old Italian Saddle Horse colt with a DSD (mosaicism; 64, XX/64, XY/63, XO). (**A**) Perineal region, ventral to the anal sphincter; a median raphe approximately 20 cm in length was present, terminating ventrally in a micropenis measuring approximately 11 × 3 cm. (**B**) Gross appearance of subcutaneous hypoplastic testes following inguinal castration. (**C**) Histological section of testis which consists of numerous seminiferous tubules reduced in diameter lined by a single layer of Sertoli cells protruding into the lumen (asterisk) separated by elongated to polygonal interstitial cells (arrowhead) (H&E, 10×).

Case 2, a 15-month-old Italian Saddle Horse colt, additionally exhibited mild enlargement of the inguinal mammary teats. Ultrasonography identified two subcutaneous testes located at the level of the external inguinal rings. Cytogenetic analysis revealed chromosomal mosaicism (64, XX/64, XY/63, XO) with a positive SRY gene (Figure 2).

**Figure 3:**
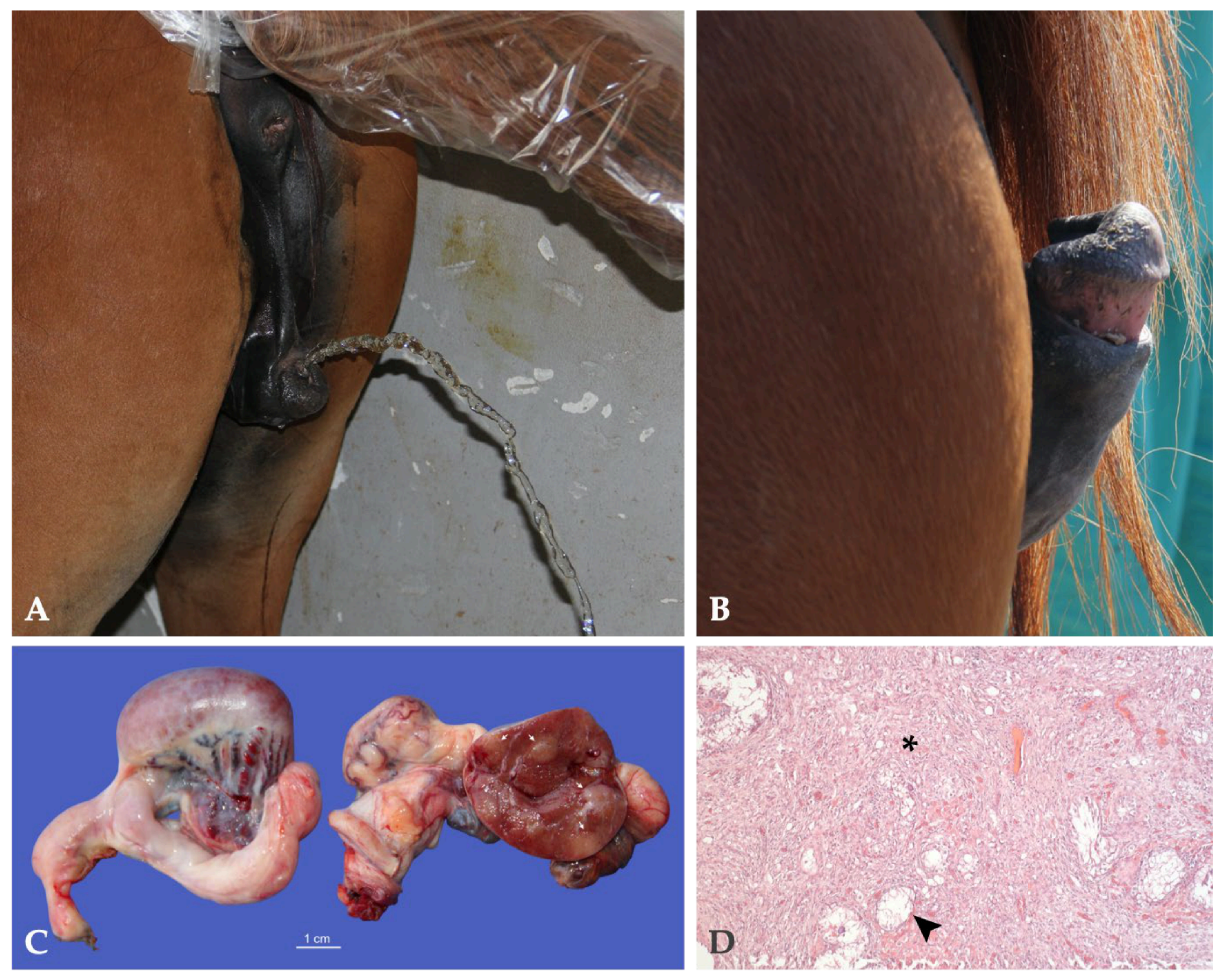
**Case 3.** Three-year-old Arabian stallion with a DSD (64, XX karyotype with a negative SRY gene). (**A**) Perineal conformation during urination, characterized by an elongated median raphe, about 20 cm, extending ventrally from the anus and terminating in a small caudally directed penis, measuring approximately 5 × 3 cm, with urine discharged in a characteristic arched stream. (**B**) Close-up view of the mushroom-shaped glans penis, representing an unusual external genital malformation. (**C**) Gross appearance of the left abdominal testis (**A**) and right inguinal testis (**B**). Arrows indicate multiple nodular proliferations corresponding to a Sertoli cell tumor. (**D**) Histological section of testis with diffuse proliferation of neoplastic Sertoli cells replacing the testicular parenchyma (asterisk) and surrounding and entrapping residual hypoplastic seminiferous tubules (arrowhead) (H&E, 10×).

Case 3, a 3-year-old Arabian stallion, also showed mild enlargement of the inguinal mammary teats and presented with a left abdominal and a right subcutaneous testis. Unlike the other horses, the right subcutaneous gonad additionally showed a Sertoli cell tumor. Cytogenetic analysis identified a 64, XX karyotype with a negative SRY gene (Figure 3).

**Figure 4:**
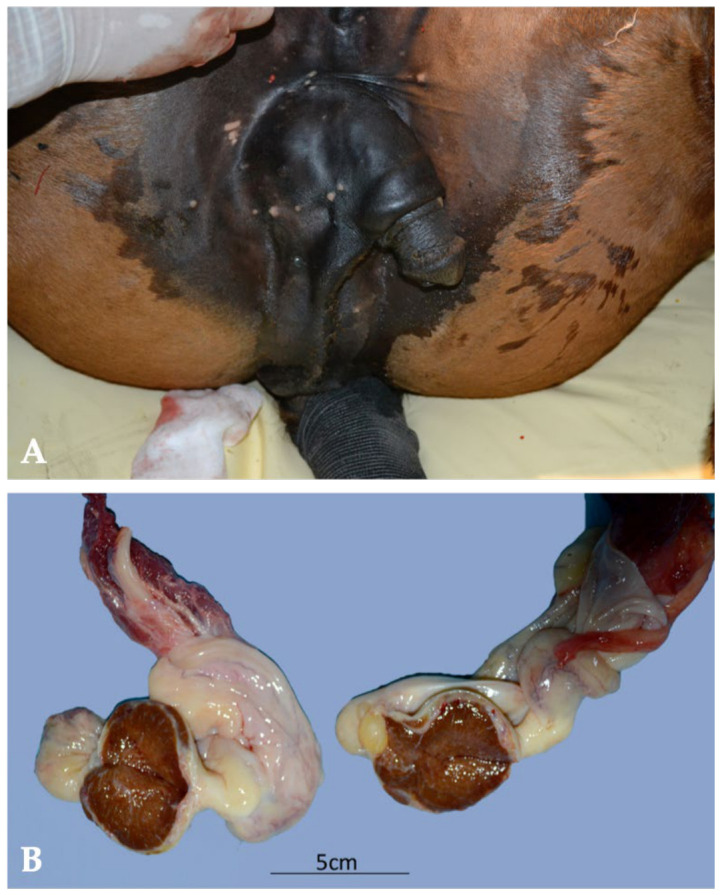
**Case 4.** (**A**) Eighteen-month-old Arabian horse with a DSD. Perineal region showing an elongated median raphe, from which a penis-like structure protruded ventrally for approximately 10 cm. (**B**) Gross appearance of the bilateral hypoplastic subcutaneous testes following orchiectomy on cut section.

Case 4, an 18-month-old Arabian horse, exhibited bilateral enlargement of the inguinal mammary teats. Ultrasonography demonstrated two subcutaneous testes. Cytogenetic analysis was not performed (Figure 4).

**Figure 5:**
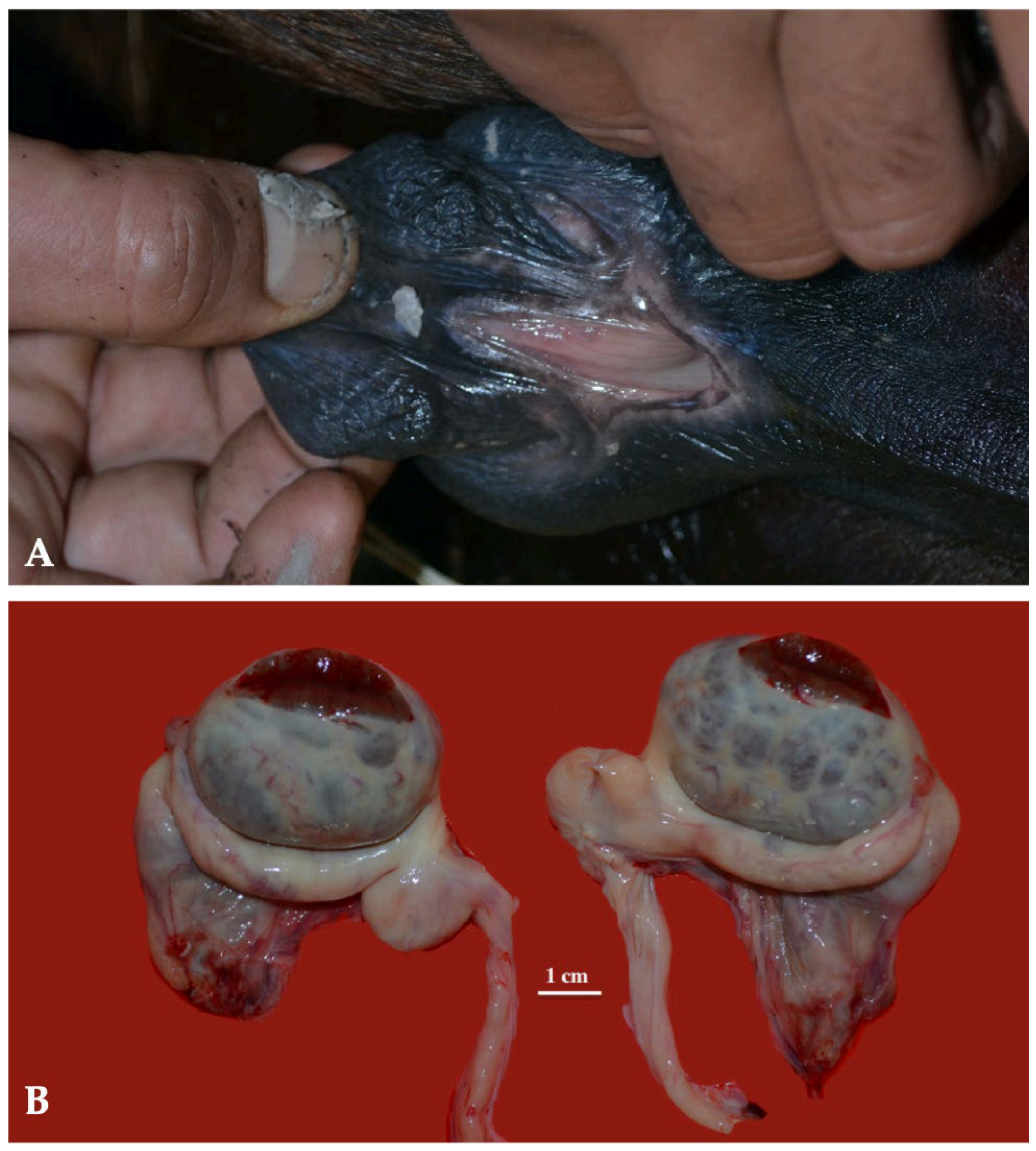
**Case 5.** Two-year-old Trotter horse with a DSD. (**A**) Epispadias malformation characterized by a dorsally displaced urethral meatus associated with abnormal penile development. (**B**) Gross appearance of bilateral subcutaneous hypoplastic testes removed during orchiectomy.

Case 5, a 2-year-old Trotter horse, differed from the other DSD cases by the presence of an unusual epispadias-like malformation characterized by a dorsally positioned urethral opening on the micropenis. Ultrasonography demonstrated two subcutaneous testes. Cytogenetic analysis was not performed (Figure 5).

**Figure 6:**
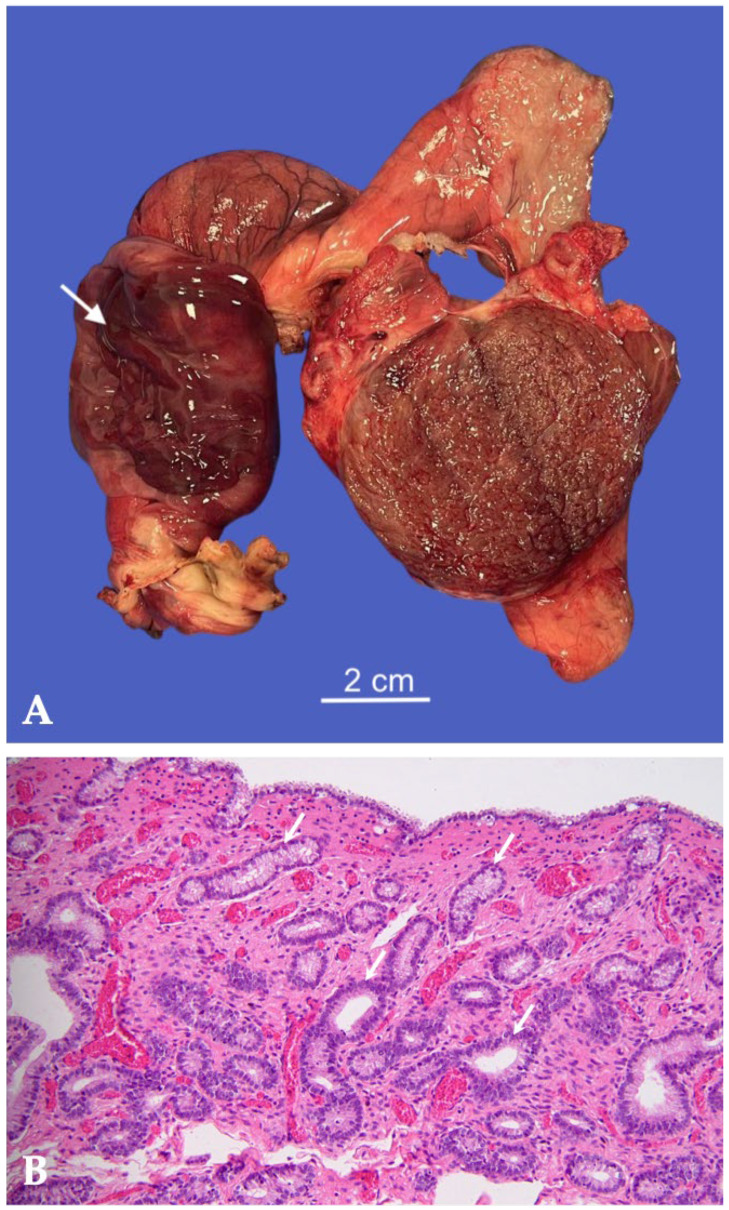
**Case 6.** Three-year-old Spanish horse with bilateral cryptorchidism. (**A**) Gross pathology showing the cut surface of the retained gonad, including the testis and epididymis, demonstrating a cavitary uterine-like structure (arrows) arising from the caudal aspect of the epididymis and replacing the expected ductus deferens. (**B**) Histological section of rudimentary uterine structure composed of well-developed endometrium with numerous endometrial glands in the corium (arrows) (H&E, 20×).

Case 6 was a 3-year-old Andalusian horse referred for bilateral cryptorchidism and represented the most unusual lesion of this series. Gross examination of one retained testis revealed a markedly enlarged tubular structure replacing the expected ductus deferens. Histopathological examination confirmed the presence of a rudimentary uterine structure characterized by a well-developed endometrial lining with multifocal endometrial glands. No normal ductus deferens structures were identified. Preliminary cytogenetic analysis demonstrated a normal male karyotype (64, XY) with a positive SRY gene (Figure 6).

#### 3.1.2. Monorchidism

Two horses were diagnosed with monorchidism. Both were referred because of suspected cryptorchidism and underwent ultrasonographic examination followed by standing laparoscopy. In both horses, laparoscopic exploration confirmed the absence of one testis, supporting the diagnosis of congenital monorchidism. Cytogenetic analysis demonstrated a normal male karyotype (64, XY) with a positive SRY gene in both cases.

Case 1, a 3-year-old Italian Heavy Draft Horse stallion, was referred for suspected right unilateral cryptorchidism. Clinical examination revealed a normally descended left testis, whereas the right gonad could not be identified by transrectal ultrasonography.

**Figure 7:**
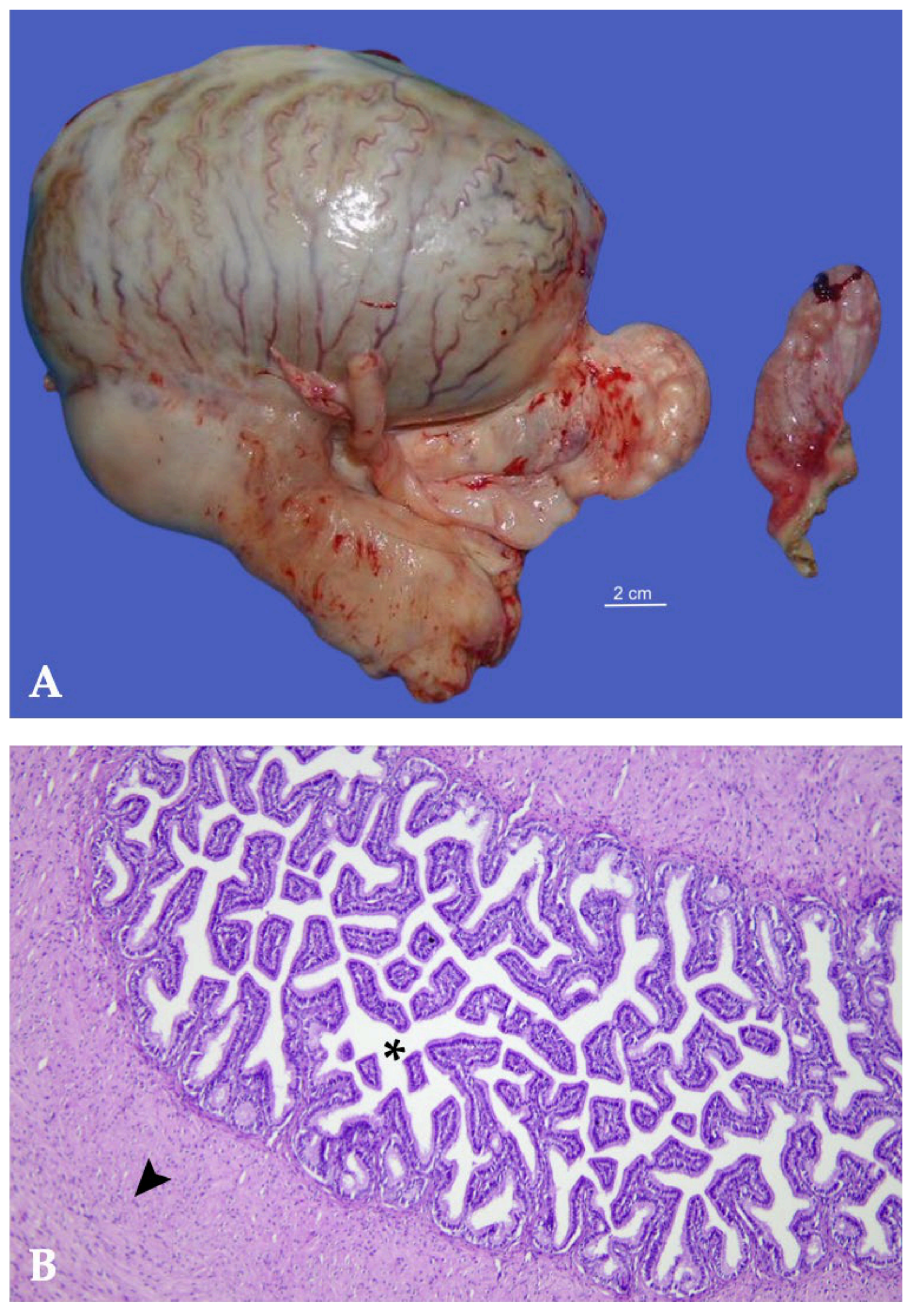
**Case 1.** Three-year-old Italian Heavy Draft Horse stallion with right-sided monorchidism. (**A**) Gross appearance of the normally descended left testis and the rudimentary ductal structures laparoscopically removed from the right side. (**B**) Histological section of testis which consists of a remnant of a ductal structure transitional between the epididymis and ductus deferens, associated with immature mesenchymal stromal tissue. Short villus-like projections lined by pseudostratified epithelium (asterisk) surrounded by a thick muscular layer (arrowhead), consistent with right-sided monorchidism (H&E, 10×).

Standing laparoscopy was therefore performed to investigate the suspected right-sided cryptorchidism. No normally developed retained testes or testicular vessels were identified. At the margin of the genital fold, a tubular structure consistent with the ductus deferens was observed, terminating in a pendulous blind-ended formation. Cranial to this structure, a second smaller pedunculated formation was identified (Figure 7A), while the internal inguinal ring appeared blind. Both structures were laparoscopically removed. The contralateral descended testis was removed using a standard open castration technique.

Histopathological examination of the left testis revealed normal parenchymal architecture with active spermatogenesis within the seminiferous tubules. The excised right-sided structures consisted of remnants of a ductal structure transitional between the epididymis and ductus deferens associated with immature mesenchymal stromal tissue (Figure 7B), while the smaller pedunculated structure identified during laparoscopy was histologically classified as immature mesenchymal tissue.

**Figure 8:**
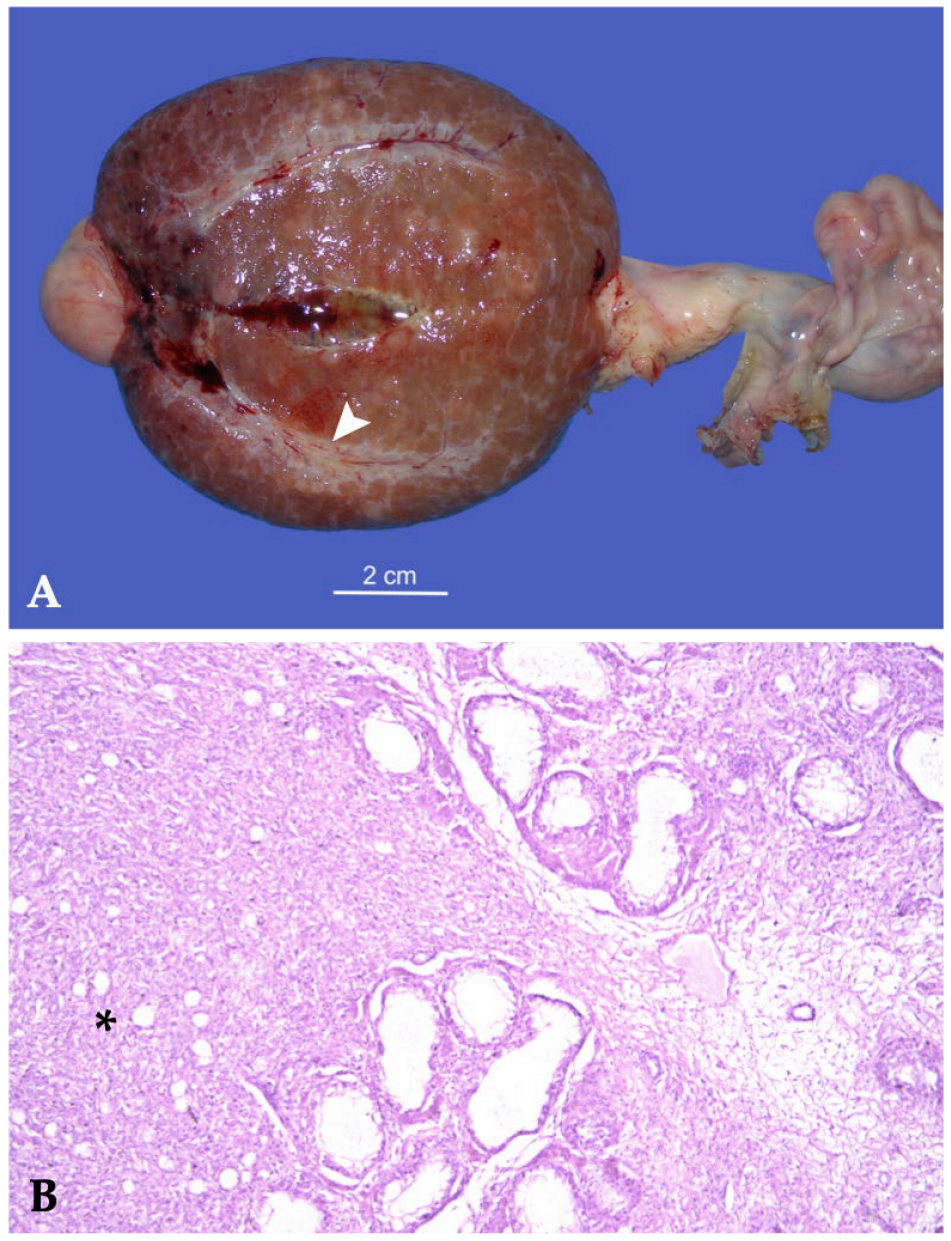
**Case 2.** Seven-year-old Appaloosa horse with right-sided monorchidism. (**A**) Gross appearance of the cut surface of the retained left abdominal testis, showing a Leydig (interstitial) cell tumor with a heterogeneous appearance due to a thin fibrous connective tissue network (arrowhead). (**B**) Histological section of testis with Leydig cell tumor. Hypertrophic vacuolated interstitial cell and fusiform cell arranged in a solid sheet (asterisk) compressing adjacent tubules (H&E, 20×).

Case 2, a 7-year-old Appaloosa horse, was referred because of the absence of scrotal testes. According to the owner, the horse had been purchased at 2 years of age, and no history of previous castration was available. Transrectal ultrasonographic examination identified a left abdominal testis measuring 7 × 4 cm, whereas the right testis could not be detected. Standing laparoscopy confirmed right-sided monorchidism. The retained left testis was removed and submitted for histopathological examination. On the right side, the mesorchium terminated against the abdominal wall adjacent to a blind inguinal ring. The genital fold exhibited a characteristic horse-head-like configuration, and its cranial margin was associated with tortuous vascular structures resembling spermatic vessels. On cut section, the retained testis showed a heterogeneous appearance due to a thin fibrous connective tissue network (Figure 8A). Histopathological examination demonstrated a well-differentiated Leydig (interstitial) cell tumor partially replacing the testicular parenchyma, with residual seminiferous tubules showing degenerative changes (Figure 3 and Figure 8B).

#### 3.1.3. Tubular Ectasia of the Rete Testis

**Figure 9:**
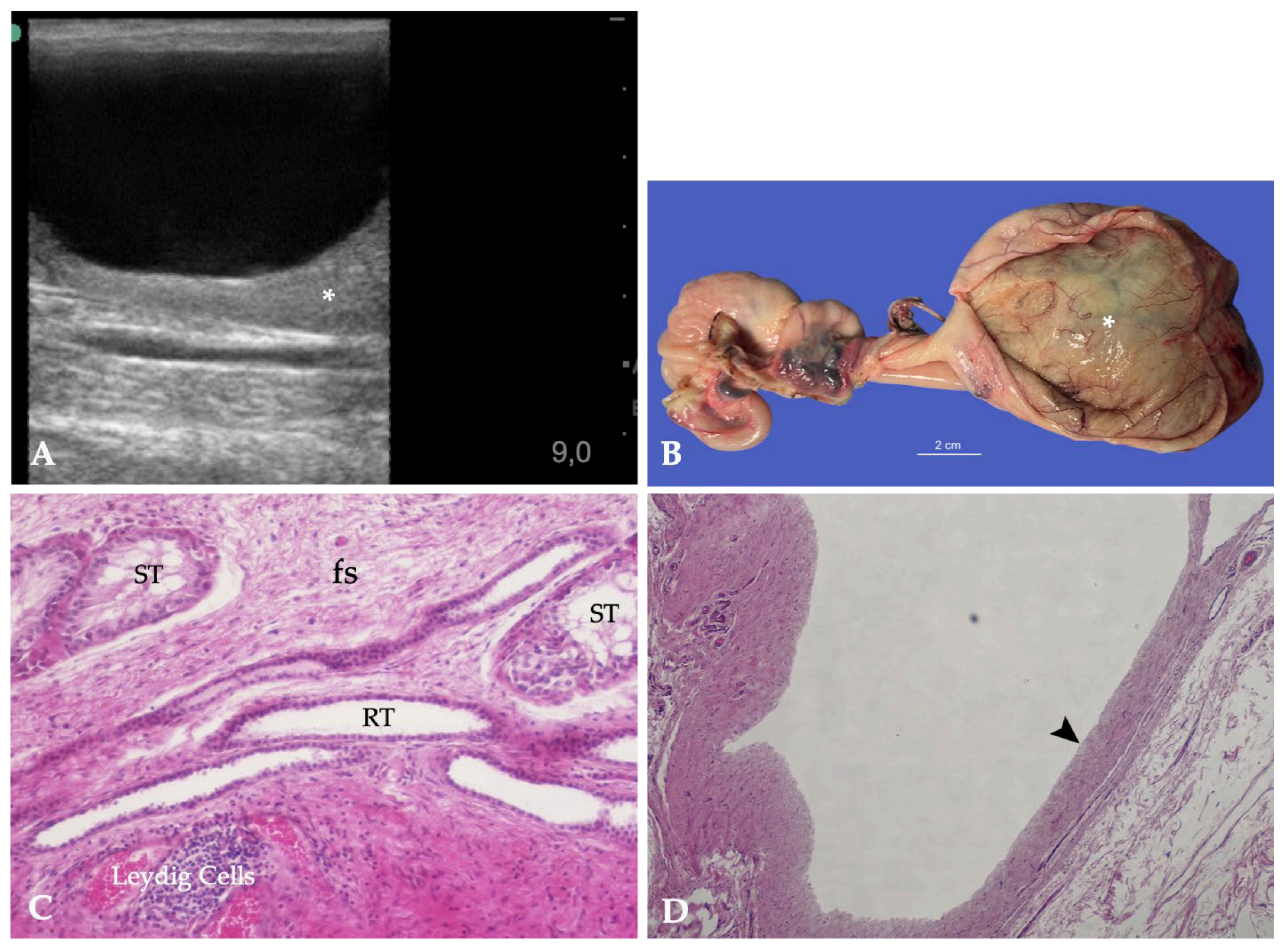
Three-year-old Arabian stallion with unilateral abdominal cryptorchidism and cystic ectasia of the rete testis. (**A**) Ultrasonographic appearance of a retained testis showing a large anechoic cystic structure measuring approximately 6 cm in diameter, surrounded by a thin rim of residual testicular parenchyma (asterisk). (**B**) Gross pathology of the retained testis showing a cut large cystic cavity occupying most of the gonadal parenchyma (asterisk). The cyst wall is thickened and smooth, with a pearly-grey inner surface. (**C**) Histopathological features of rete testis ectasia. Moderately dilated rete testis (RT) lined by a single layer of simple cuboidal epithelium. Adjacent atrophic seminiferous tubules (ST) are trapped between the fibrous stroma (fs) (H&E, 20×). (**D**) Marked cystic dilation of the rete testis, forming a large cavity lined by flattened to low cuboidal epithelium (arrowhead) and supported by a dense fibrocollagenous wall (H&E, 10×).

A 3-year-old unilaterally cryptorchid Arabian stallion was presented for elective castration. According to the owners, only the right testis had been present within the scrotum since birth. External palpation of the left inguinal ring failed to identify either the left testis or the epididymis. Transrectal ultrasonography revealed an enlarged retained testis, characterized by a preserved vascular pattern of the spermatic cord vessels, a hyperechoic tunica albuginea, and a 6 cm diameter anechoic cyst surrounded by a thin rim of testicular parenchyma (Figure 9A). A standing laparoscopic cryptorchidectomy was performed. Macroscopically (Figure 9B), the retained testis was grey-yellow in color, enlarged (9 × 7 cm), and laterally flattened. A 6 × 5 cm cyst was observed protruding from the testicular surface. The cyst wall was approximately 2 mm thick and contained 105 mL of yellow-clear fluid; the inner lining was pearlescent grey and smooth. The epididymis appeared grossly unremarkable and maintained the typical loose anatomical relationship with the testis commonly observed in cryptorchid horses. Histological (Figure 9C,D) examination revealed cystic dilation of the rete testis, which was variably sized, fluid-filled, and compressed the surrounding testicular parenchyma. Seminiferous tubules were markedly reduced in number and size, exhibiting severe atrophy. The final histopathologic diagnosis was cystic ectasia of the rete testis.

#### 3.1.4. Ectopic Adrenal Tissue

**Figure 10:**
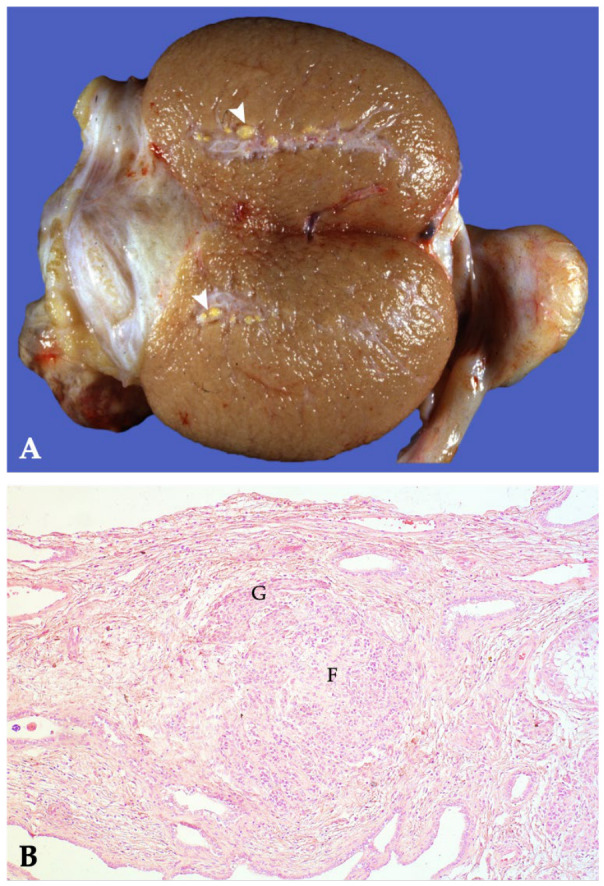
Three-year-old Trotter stallion with testicular adrenocortical cell nodules. (**A**) Gross appearance of multiple small, well-demarcated yellow nodules distributed along the course of the central testicular vein and protruding above the cut surface of the testicular parenchyma (arrowhead). (**B**) Histopathology showing nests of ectopic adrenal cortical tissue within the testicular parenchyma, displaying an irregular arrangement of zona glomerulosa-like (G) and zona fasciculata-like (F) cells (H&E, ×20).

A 3-year-old male Trotter underwent routine castration. Gross examination of the excised testes revealed multiple nodular lesions within the left testis, distributed along the course of the central vein. The nodules varied in size, ranging from 2 to 4 mm in diameter. The nodules appeared round, well-circumscribed, and yellowish in color, and were raised above the cut surface of the testicular parenchyma (Figure 10A). The left testis measured 9.5 × 6.5 cm and was considered within normal limits. On cut section, the testicular parenchyma slightly protruded beyond the tunica albuginea. No gross abnormalities of the surrounding testicular tissue were observed.

Histopathological examination demonstrated encapsulated nests and lobules of ectopic adrenal cortical tissue located in the testicular parenchyma. The ectopic tissue consisted predominantly of cells morphologically consistent with the zona fasciculata (Figure 10B). No evidence of atypia, hyperplasia, or neoplastic proliferation was identified in the adjacent testicular parenchyma, and spermatogenesis was preserved. Based on the gross and histopathological findings, a diagnosis of ectopic adrenal cortical tissue within the left testis was established.

### 3.2. Degenerative Disorders

#### 3.2.1. Ischemic Necrosis Following Failed Laparoscopic Castration Without Orchiectomy

**Figure 11:**
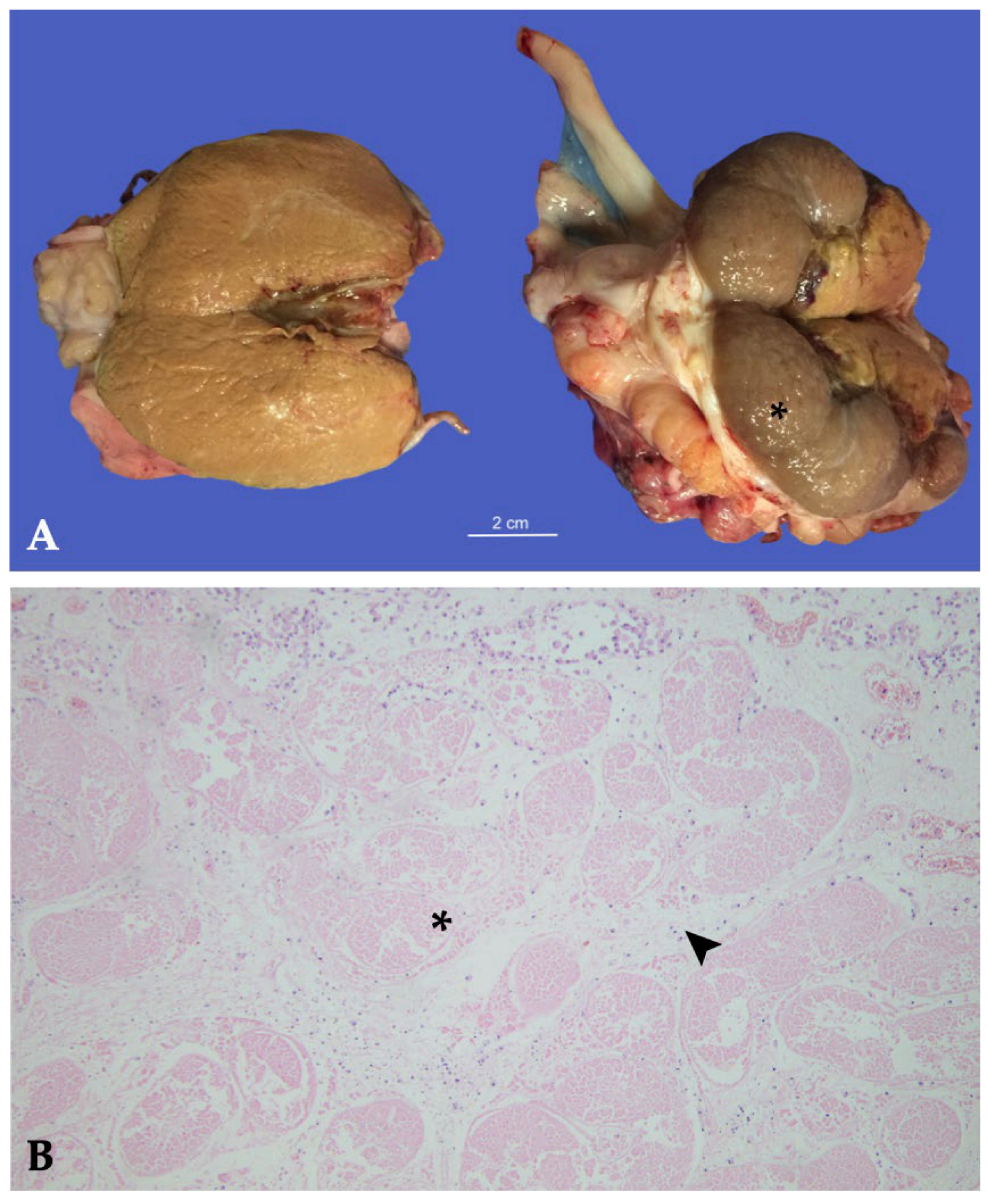
(**A**) Gross pathology of the left testis which was diffusely necrotic and reduced in size (**left**), whereas the right testis showed partial revascularization with central necrosis and peripheral viable parenchyma (asterisk) (**right**). (**B**) Histological section of testis composed of hypereosinophilic lobules with loss of nuclei (asterisk) separated by expanded mildly inflamed interstitium (arrowhead) (H&E, 10×).

Two cases of ischemic testicular necrosis following failed laparoscopic castration without orchidectomy were observed in one mixed-breed 5-year-old horse and in one 6-year-old heavy draft horse. In these two horses, subjected to laparoscopic castration without orchiectomy, the condition was expected to result in avascular necrosis of the testis. However, three months after laparoscopic surgery, these horses exhibited stallion-like behavior due to revascularization of one testis. These horses were subsequently castrated with a standard closed technique. On gross examination, the necrotic testis appeared markedly reduced in size, with a dull and yellowish color (Figure 11A). The testis that was revascularized presented partially central necrotic parenchyma and a peripheral area of normal, prominent testicular parenchyma, responsible for testosterone secretion and the persistence of stallion-like behavior. Histopathological examination of the testis that appeared grossly necrotic revealed diffuse coagulative necrosis involving the entire parenchyma (Figure 11B). The necrotic process affected both the germinal epithelium of the seminiferous tubules and the gonadal stromal–interstitial cells. Multifocally, at the periphery of the testicular tissue, the interstitium was mildly expanded by inflammatory infiltrates composed predominantly of eosinophils, mixed with rare lymphocytes and macrophages, consistent with moderate eosinophilic periorchitis. In contrast, the contralateral testis, which appeared partially viable on gross examination, exhibited a heterogeneous histological pattern. Locally extensive areas of coagulative necrosis, similar to those observed in the completely necrotic testis, were interspersed with areas of preserved seminiferous tubules showing active spermatogenesis, characterized by spermatocytes at various stages of maturation. Other tubules displayed moderate degeneration of the seminiferous epithelium, with reduced numbers of spermatocytes, rare spermatids, and degenerated Sertoli cells exhibiting marked cytoplasmic vacuolation, consistent with testicular degeneration. Multifocally, the epididymal ducts contained abundant mature spermatozoa. Moderate eosinophilic inflammatory infiltrates were also present at the periphery of the necrotic areas.

#### 3.2.2. Bilateral Testicular Degeneration Associated with Anabolic–Androgenic Steroid Abuse

A 15-year-old Thoroughbred stallion was referred for evaluation of azoospermia and infertility. According to the clinical history, the horse had previously been used for athletic activity, and prolonged administration of anabolic-androgenic steroids (AASs) was suspected.

**Figure 12:**
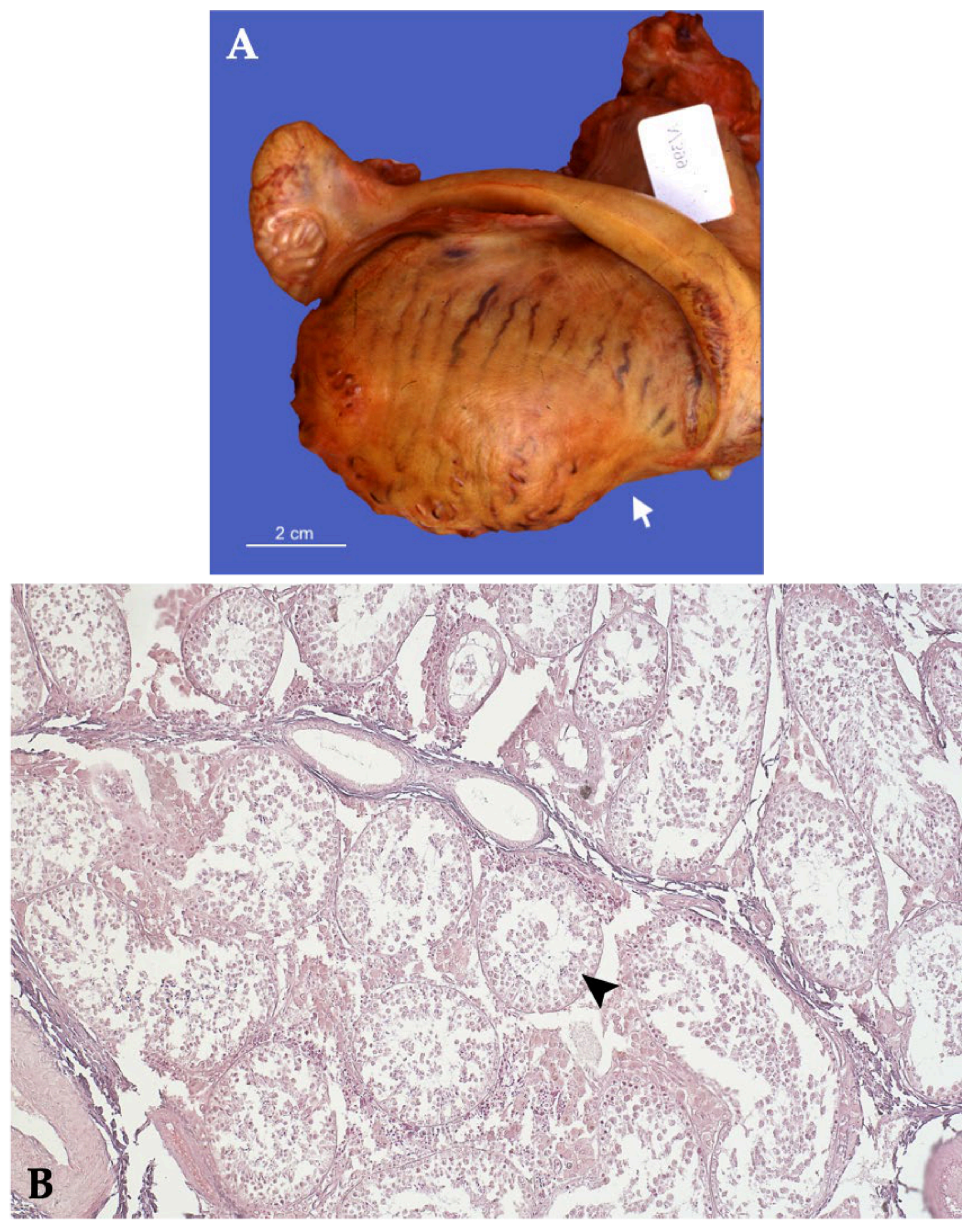
(**A**) Gross appearance of a markedly reduced testis showing focal depressed area (arrow). The tunica albuginea is diffusely thickened and fibrotic. (**B**) Histopathology showing diffuse degeneration and atrophy of the seminiferous tubules. Tubules consist of rare spermatids and mature germ cells and numerous Sertoli cells (arrow) (H&E, 20×).

Clinical and reproductive examination revealed bilateral reduction in testicular size and consistency. The stallion subsequently underwent bilateral castration using a standard closed technique. Gross examination revealed both testes to be markedly reduced in volume, with a focal depressed area, consistent with loss of testicular parenchyma. The tunica albuginea appeared diffusely thickened and fibrotic (Figure 12A).

Histopathological examination demonstrated diffuse degeneration and atrophy of the seminiferous tubules (Figure 12B). The seminiferous tubules were markedly reduced in diameter and depleted of germinal epithelium, with severe reduction or complete absence of spermatids and mature germ cells. In several tubules, only Sertoli cells remained evident. The interstitial compartment showed marked depletion and advanced atrophy of Leydig cells. Based on the clinical history, gross findings, and histopathological features, a diagnosis of bilateral irreversible testicular degeneration associated with suspected anabolic steroid-induced hypogonadism was established.

#### 3.2.3. Spermatic Cord Torsion

Three horses were diagnosed with spermatic cord torsion (Table 2). One horse presented with chronic torsion involving an abdominal retained testis, whereas the remaining two horses were referred because of acute unilateral scrotal enlargement and severe colic pain associated with torsion of a descended testis. In scrotal cases, ultrasonography demonstrated marked vascular compromise of the affected gonad, and the diagnosis was confirmed during surgery and histopathological examination.

**Figure 13:**
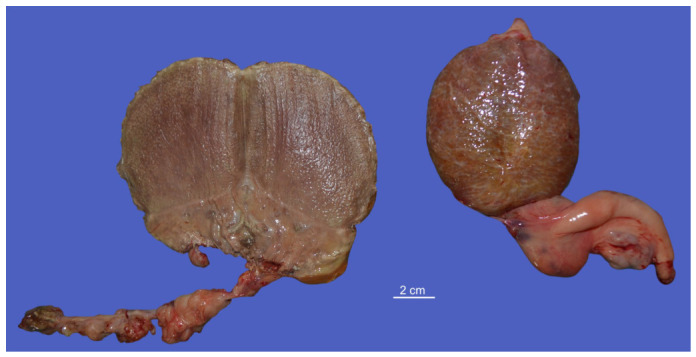
**Case 1.** Six-year-old Quarter Horse stallion with chronic torsion (>360°) of a retained left abdominal testis. Gross appearance of the bilateral cryptorchid testes. The left testis appeared soft and discolored (**left**), with a characteristic peanut butter-like appearance and complete loss of recognizable epididymal structures. Contralateral retained abdominal testis (**right**).

Case 1, a 6-year-old Quarter Horse stallion, was admitted for bilateral cryptorchidectomy. Preoperative transrectal ultrasonography identified the right retained testis, whereas the left testis could not be visualized. During standing laparoscopic cryptorchidectomy, the left abdominal testis was identified adjacent to the urinary bladder and intestinal loops. The spermatic cord showed torsion exceeding 360°, with complete absence of detectable blood flow. According to the owner, the horse had never exhibited signs of colic or abdominal discomfort, despite having been maintained at pasture under extensive management. On macroscopic examination (Figure 13), the left testis measured 9 × 7 × 2.5 cm and appeared soft and discolored, with a peanut butter-like color, and no recognizable epididymal structures were identified. Similar degenerative alterations were observed in the tunica albuginea, while the spermatic cord appeared markedly thinned and almost filiform. Histopathological examination demonstrated severe diffuse degeneration and necrosis of the seminiferous tubules, consistent with chronic vascular compromise.

**Figure 14:**
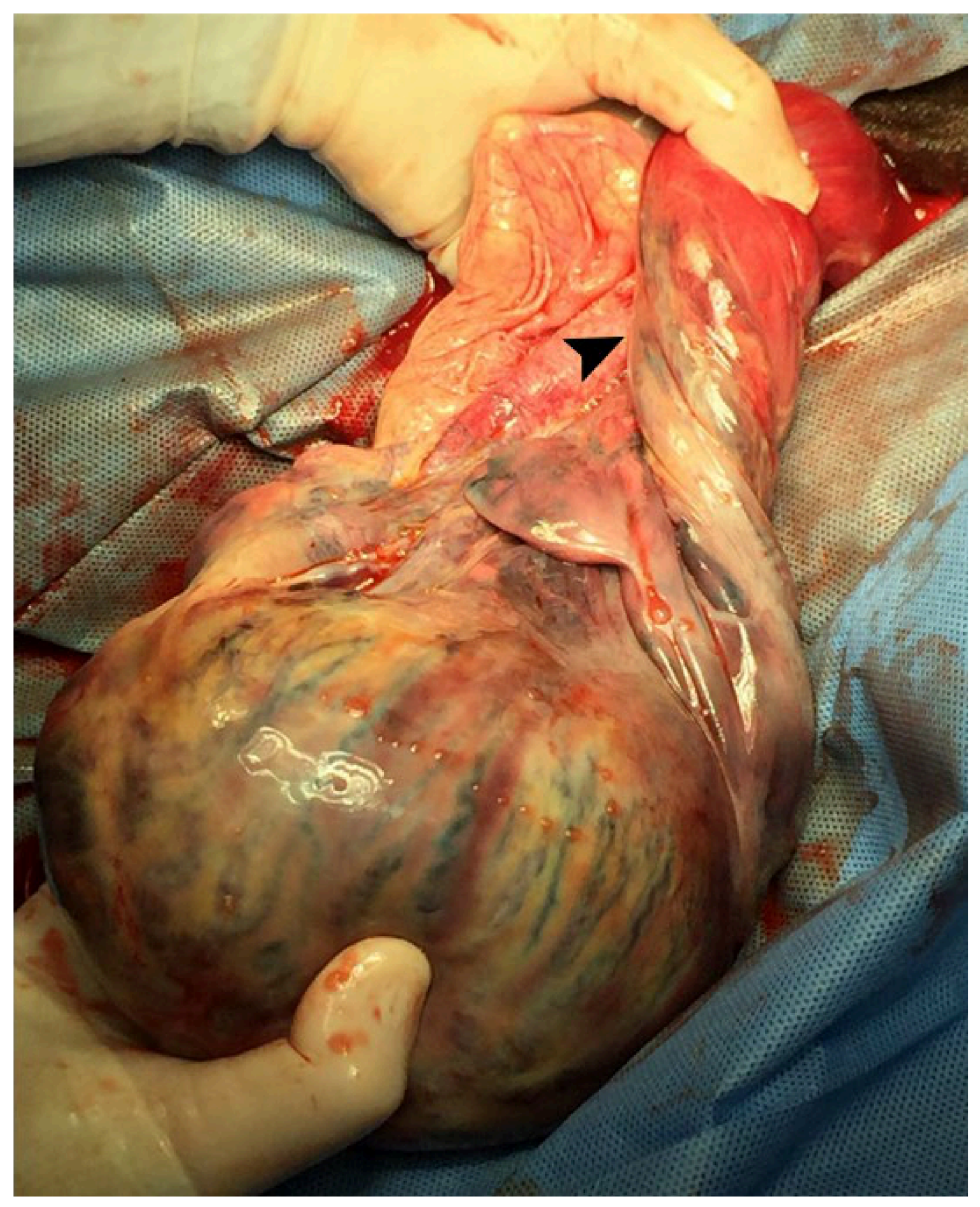
**Case 2.** Intraoperative photograph of approximately 270° spermatic cord torsion in a stallion following exteriorization of the affected testis. The spermatic cord is rotated approximately three-quarters of a full turn, resulting in marked venous outflow obstruction with severe vascular congestion (arrowhead). The testis is markedly enlarged and diffusely dark red to purple, with a smooth but tense tunica vaginalis and multifocal subcapsular ecchymoses. Prominent, congested vessels are visible along the spermatic cord and vaginal tunic, consistent with impaired venous drainage and progressive ischemic injury.

Cases 2 and 3 were referred because of acute spermatic cord torsion associated with acute scrotal enlargement. Case 2, a 12-year-old Missouri Fox Trotter stallion used for competitive trail riding, endurance, and breeding, was referred to the Veterinary Teaching Hospital for evaluation of colic associated with acute right scrotal enlargement. Case 3, a 5-year-old Trotter stallion, was referred for acute onset of severe pain associated with enlargement of the left hemiscrotum. In both horses, physical examination revealed marked enlargement of the affected testis, which was firm and painful on palpation, whereas the contralateral testis was unremarkable. In Case 2, the horse was bright, alert, and responsive, with mildly hyperemic mucous membranes, a subtle toxic line, and multifocal petechiation. The right testis was extremely firm, closely opposed to the body wall, and not freely movable within the scrotum, whereas the left testis was soft and freely movable. In Case 3, a mild hydrocele was additionally present within the affected hemiscrotum (Figure 14).

Ultrasonographic examination demonstrated marked reduction in intratesticular blood flow in both horses, associated with severe vascular compromise of the affected gonad. In Case 2, the right spermatic cord showed reduced blood flow and thickening of the testicular arterial wall, while the central vein appeared mildly dilated (approximately 4.5 mm in diameter) without detectable blood flow. The testicular parenchyma showed a heterogeneous mottled appearance with marked disruption of intraparenchymal blood flow. Similarly, in Case 3, ultrasonography demonstrated enlargement of the left spermatic cord, mild hydrocele, and diffuse heterogeneous alteration of the testicular parenchyma, consistent with vascular compromise secondary to acute spermatic cord torsion. Venous obstruction resulted in severe congestion, progressive ischemic injury, and testicular necrosis.

Surgical treatment differed between the two horses. Case 2 underwent unilateral castration of the affected right testis using a semi-closed castration technique (Figure 14), whereas Case 3 underwent bilateral castration using a standard closed castration technique.

**Figure 15:**
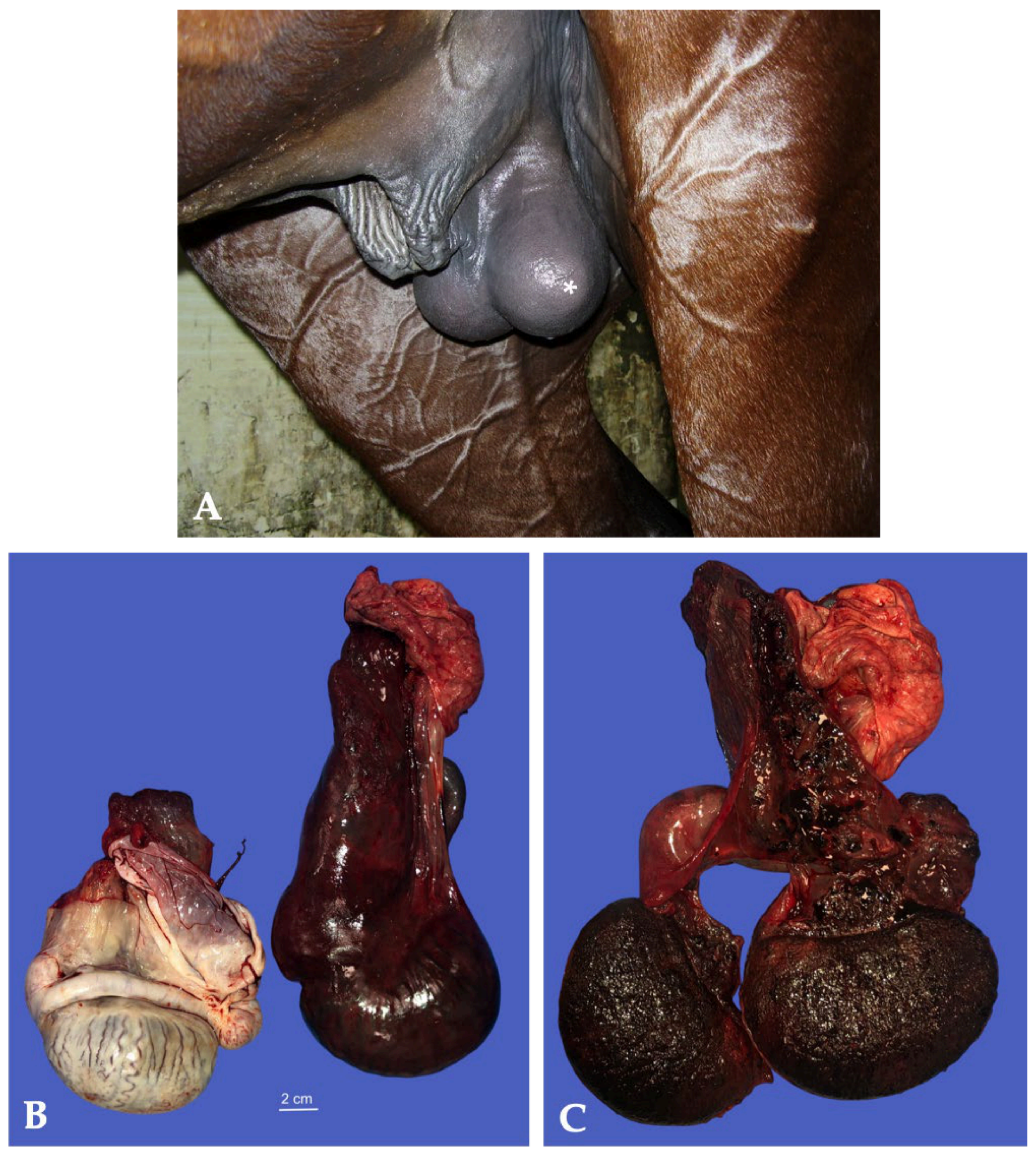
**Case 3.** Five-year-old Trotter stallion with acute left spermatic cord torsion (>420°). (**A**) Clinical appearance of the enlarged left hemiscrotum, the asterisk indicates cranial displacement of the epididymal tail, a characteristic finding associated with spermatic cord torsion. (**B**) Gross appearance of the left testis. Compared with the previous case, note the marked dark discoloration of the testis, reflecting severe vascular and parenchymal compromise consistent with spermatic cord torsion exceeding 420°. (**C**) Cut surface of the affected testis showing diffuse intravascular coagulation.

Gross examination of the affected testes revealed similar lesions in both horses, characterized by marked enlargement and severe hemorrhagic vascular compromise. In Case 2, the right testis exhibited a lobulated dark-purple surface, and the testis, epididymis, and associated testicular vasculature appeared devitalized. On sectioning, the testicular parenchyma showed a dark red-to-black hemorrhagic and necrotic appearance, consistent with severe vascular compromise and infarction. In Case 3, the left testis showed diffuse red-brown discoloration involving the entire parenchyma, which appeared diffusely congested and necrotic on cut section (Figure 15).

Histopathological examination demonstrated comparable lesions in both horses, consisting of severe diffuse vascular congestion, interstitial hemorrhage, acute coagulative necrosis of the seminiferous tubules, multifocal vascular thrombosis, and edema, consistent with acute hemorrhagic infarction secondary to spermatic cord torsion.

#### 3.2.4. Compensatory Hypertrophy and Hyperplasia in Retained Testes

Twenty-three horses and one pony were referred with a history of previous castration performed in field conditions and were subsequently evaluated because of persistent stallion-like behavior. Laparoscopic exploration, performed 2 to 3 years after castration of the scrotal testis, revealed retained abdominal testes, confirming incomplete castration. Standing laparoscopic cryptorchidectomy was subsequently performed in all cases. In all cases, the remaining retained testes showed marked compensatory enlargement, characterized by hyperplasia and hypertrophy of the testicular parenchyma.

**Figure 16:**
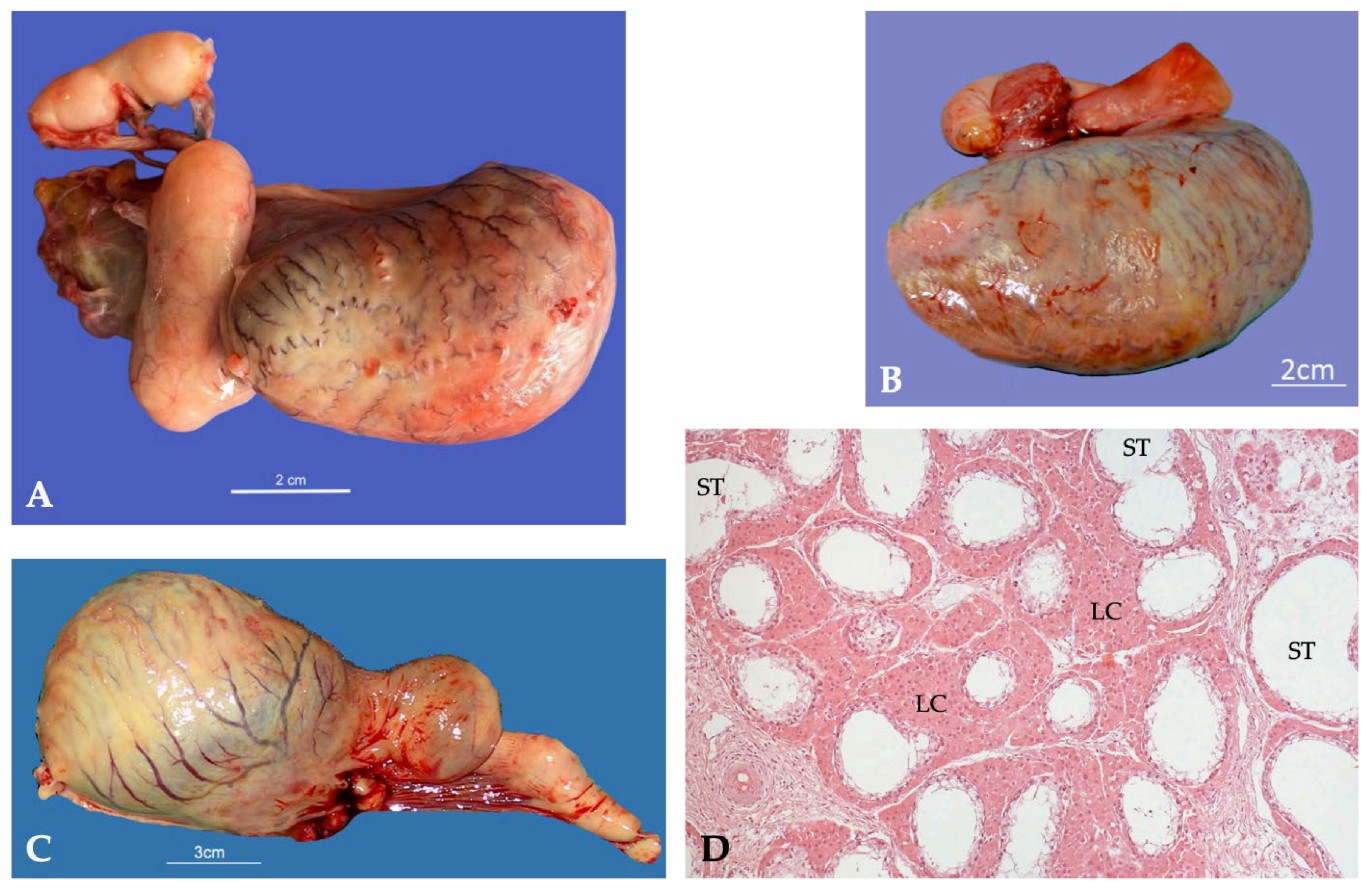
(**A**) Four-year-old pony with compensatory hypertrophy and hyperplasia of a retained left cryptorchid testis, evaluated two years after hemicastration of the right scrotal testis. The retained testis measured 7 × 4.5 cm. (**B**) Five-year-old Quarter Horse stallion with compensatory hypertrophy and hyperplasia of a retained right cryptorchid testis following incomplete castration. The retained testis measured 11 × 6 cm. (**C**) Five-year-old Appaloosa stallion with compensatory hypertrophy and hyperplasia of a retained cryptorchid testis, evaluated three years after unilateral castration of the contralateral scrotal testis. The retained testis measured 9.5 × 7 cm. (**D**) Histological section of retained testis characterized by marked expansion of the interstitial compartment due to hypertrophy and hyperplasia of the interstitial (Leydig) cells (LC) and compression of adjacent seminiferous tubules (ST) (H&E, 20×).

Grossly, the retained testes were consistently enlarged (Figure 16A–C). Histopathological examination revealed compensatory hypertrophy and hyperplasia of the retained cryptorchid testes, characterized predominantly by marked expansion of the interstitial compartment due to Leydig cell hyperplasia and hypertrophy, with less prominent involvement of the seminiferous tubules (Figure 16D).

#### 3.2.5. Intratesticular Cyst

A 6-year-old Saddlebred stallion was referred for elective cryptorchidectomy. Clinical examination revealed a normally descended left testis within the scrotum, whereas the right testis was retained within the abdominal cavity. Standing laparoscopic cryptorchidectomy of the retained right testis and routine castration of the contralateral scrotal testis were performed.

**Figure 17:**
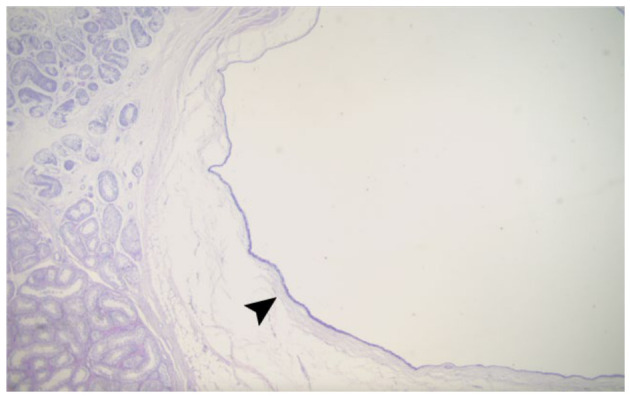
Histological section of the testis with a large, unilocular empty cavity lined by a single layer of cuboidal or flattened epithelial cells (arrow) (H&E, 20×).

Gross examination of the left eutopic testis, which was considered normal in size and appearance, revealed an incidental cystic lesion measuring approximately 2 cm in diameter. The cyst was located immediately beneath the tunica albuginea, between the tunica and the testicular parenchyma, and contained a clear serous fluid. The lesion did not alter the external contour of the testis and was not associated with any gross abnormalities of the adjacent parenchyma. The sub-albuginea cyst was sampled for histopathological examination, which revealed a large, unilocular, empty cavity lined by a single layer of cuboidal or flattened epithelial cells (Figure 17).

### 3.3. Inflammatory and Parasitic Disorders

#### 3.3.1. Hydrocele and Periorchitis

Four horses were diagnosed with hydrocele and chronic inflammatory disorders of the vaginal cavity (Table 3). One horse presented with a simple hydrocele, whereas the remaining three horses had recurrent hydroceles following ultrasound-guided aspiration performed under field conditions. Progressive recurrence was associated with increasing organization of the vaginal contents, development of multiple septations, and chronic inflammatory changes involving the vaginal tunics and testicular parenchyma.

Case 1, an 11-year-old Italian Saddle horse, was referred for evaluation of an enlargement of the right hemiscrotum. Ultrasonographic examination demonstrated a hydrocele characterized by accumulation of anechoic fluid within the vaginal cavity surrounding the testis. Bilateral orchiectomy was performed.

**Figure 18:**
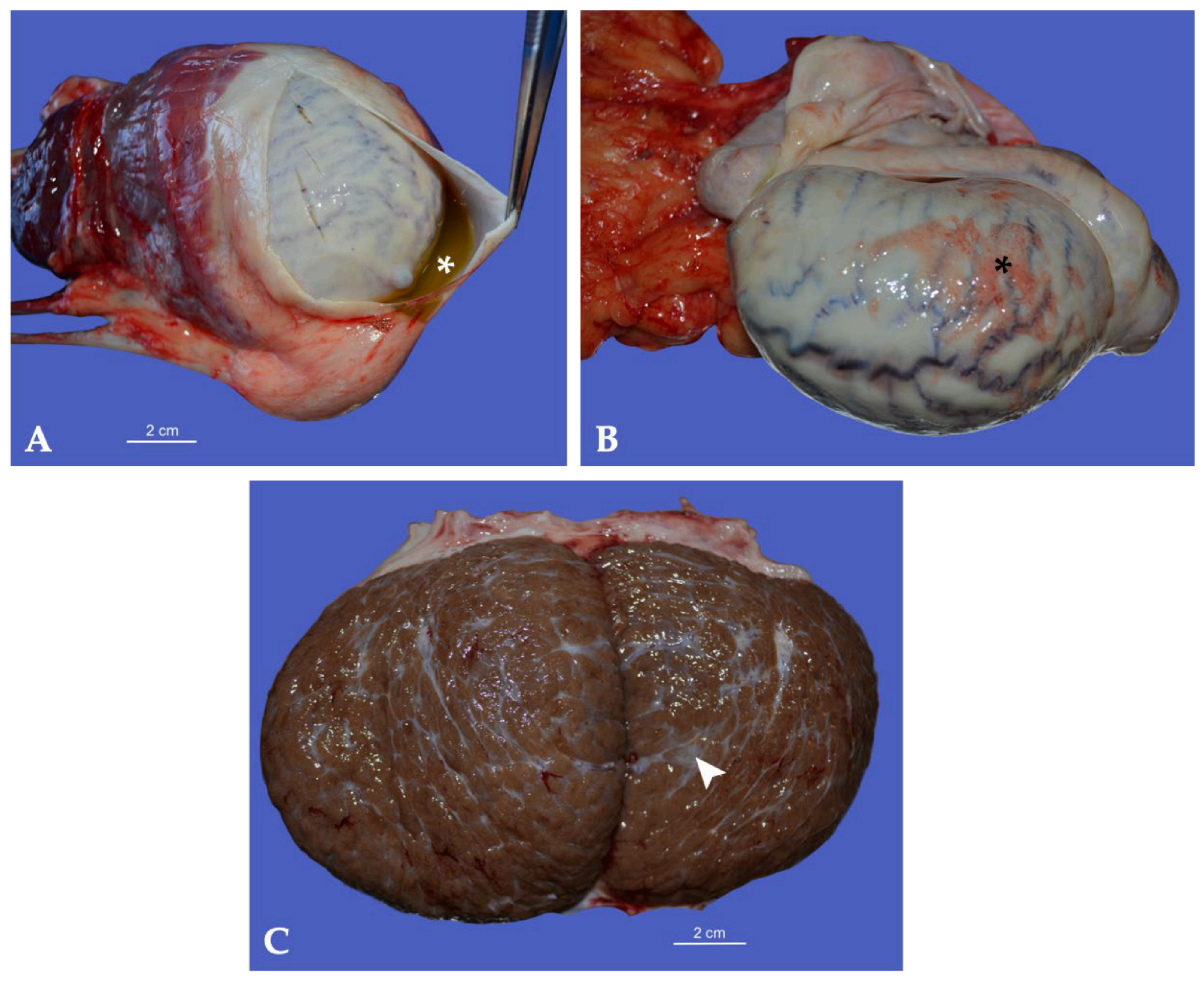
**Case 1.** Eleven-year-old Italian Saddle Horse with hydrocele. (**A**) Gross appearance of the affected testis showing mild distension of the vaginal cavity due to accumulation of yellowish gelatinous fluid (asterisk) and diffuse thickening of the common vaginal tunic. (**B**) Gross appearance showing diffuse thickening of the tunica vaginalis, appearing intensely white, with a diffuse rugose surface and adherent pinkish-yellow fibrinous exudate (asterisk) on the tunica vaginalis and vascular ectasia on the free margin. (**C**) Cut surface of the affected testis, showing fibrous septations extending throughout the testicular parenchyma (arrowhead).

Gross examination revealed a mild distension of the vaginal cavity due to the presence of yellowish gelatinous fluid (Figure 18A). The tunica vaginalis was diffusely thickened and opaque. The surface exhibited a diffuse rugose appearance, with widespread deposits of pinkish-yellow fibrinous exudate firmly adhering to the tunica vaginalis (Figure 18B).

On cut section, the affected testis showed a darker brown coloration and was traversed by fibrous septa, findings consistent with chronic degenerative changes in the testicular parenchyma (Figure 18C).

Cases 2–4 shared a similar clinical history. All horses initially presented with a non-painful hydrocele that had been treated by ultrasound-guided aspiration under field conditions, from referring veterinarians, followed by antimicrobial and anti-inflammatory therapy. Despite an initial reduction in scrotal size, rapid recurrence occurred within a few months, with progressive enlargement of the affected hemiscrotum and increasing ultrasonographic complexity. Repeat ultrasonographic examination demonstrated evolution from a simple hydrocele to a complex septate lesion characterized by multiple honeycomb-like fluid-filled compartments, diffuse thickening of the tunica albuginea, and heterogeneous echogenicity of the testicular parenchyma. Surgical treatment consisted of orchiectomy.

Case 2 and 3, a 5-year-old Spanish horse and a 7-year-old Friesian, were referred to the hospital by veterinary practitioners for evaluation of progressive enlargement of the left hemiscrotum. As neither horse had breeding value and the owners wished to avoid castration as an initial treatment option, the referring veterinarians performed ultrasound-guided aspiration of the hydrocele fluid. The aspirated fluid was clear and serous, and both horses received medical treatment following drainage.

Repeat ultrasonographic examination, performed at the Veterinary Teaching Hospital, demonstrated progression from a simple hydrocele to a “complex” septate lesion characterized by multiple honeycomb-like fluid-filled compartments surrounding the testis. The testicular parenchyma appeared reduced in size, with heterogeneous echogenicity, multifocal hyperechoic areas, and diffuse thickening of the tunica albuginea. Owing to the rapid recurrence and progression of the lesions, bilateral orchiectomy of the affected testis was subsequently performed under general anesthesia using a standard closed technique in both horses.

**Figure 19:**
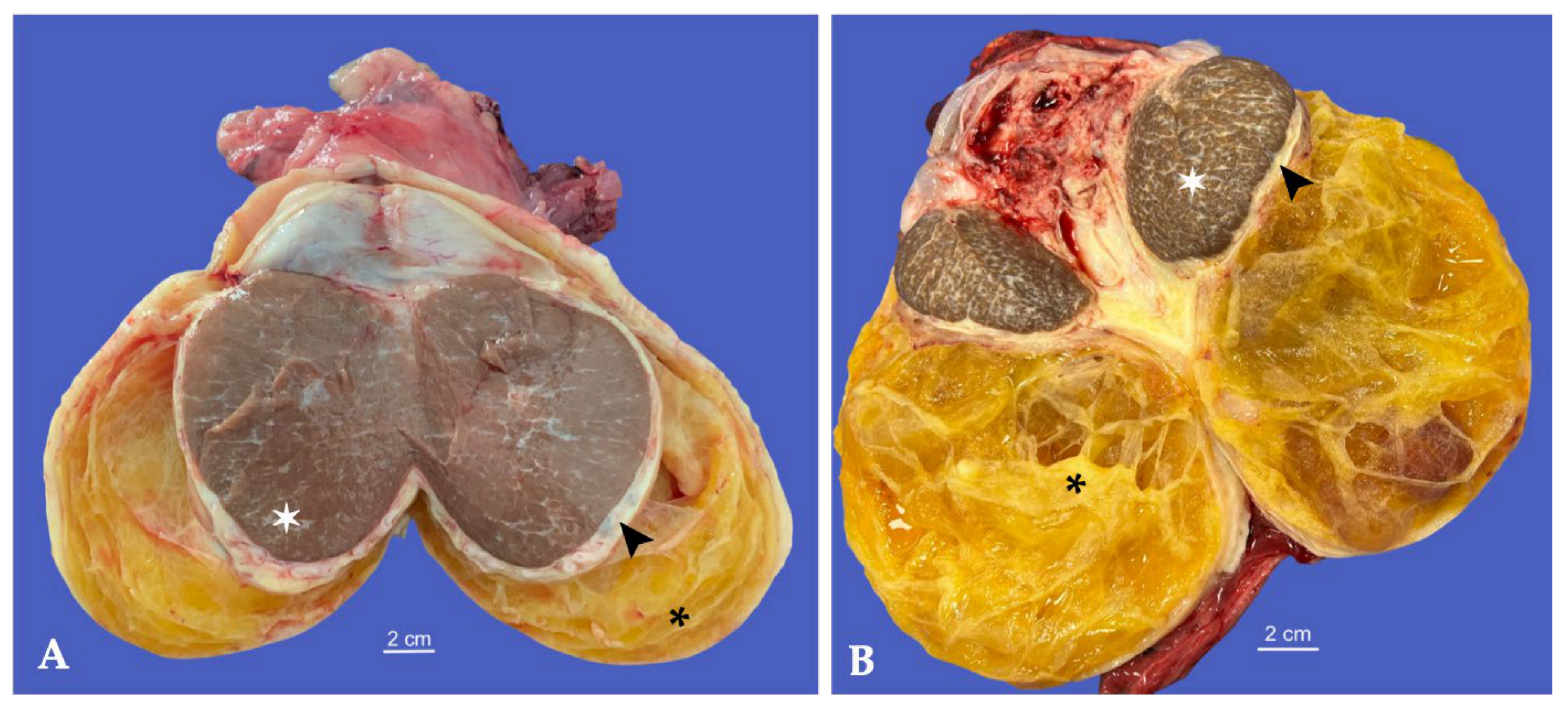
(**A**) **Case 2.** Five-year-old Spanish Horse with chronic periorchitis following recurrence of a hydrocele after ultrasound-guided aspiration. Gross appearance of the affected testis showing marked enlargement of the vaginal cavity containing abundant yellowish gelatinous material with multiple septations and organized serofibrinous exudate (asterisk). Diffuse thickening of the tunica albuginea (arrowhead) and parietal tunica vaginalis, together with multifocal fibrous septa within the testicular parenchyma (star), are evident. (**B**) Case 3. Seven-year-old Friesian stallion with chronic periorchitis following recurrence of a hydrocele after ultrasound-guided aspiration. Gross appearance of the affected testis showing a markedly enlarged vaginal cavity (asterisk) containing abundant organized serofibrinous material with numerous fibrous. Marked thickening of the tunica albuginea septa (arrowhead) and diffuse fibrosis of the testicular parenchyma (star) are evident.

In the Spanish horse, gross examination revealed a markedly enlarged vaginal cavity measuring 15 × 12 × 8 cm and containing abundant yellowish gelatinous material with multiple septations and organization of serofibrinous exudate. The tunica albuginea and parietal tunica vaginalis were diffusely thickened. The testis measured 12 × 9 cm and was firm, with multifocal whitish fibrous septations extending throughout the parenchyma (Figure 19A).

In the Friesian horse, gross examination revealed a markedly enlarged vaginal cavity measuring 24 × 14 × 9 cm and containing abundant organized serofibrinous material with numerous fibrous septa. The tunica albuginea was markedly thickened, measuring approximately 8 mm. The testis measured 8 × 6.5 cm and was diffusely firm, with multiple whitish fibrous bands extending throughout the parenchyma, consistent with extensive fibrosis (Figure 19B).

Based on the clinical history, ultrasonographic findings, recurrence following hydrocele aspiration, and gross pathological features, a presumptive diagnosis of chronic periorchitis with inflammation of the vaginal cavity secondary to hydrocele drainage was made in both horses.

**Figure 20:**
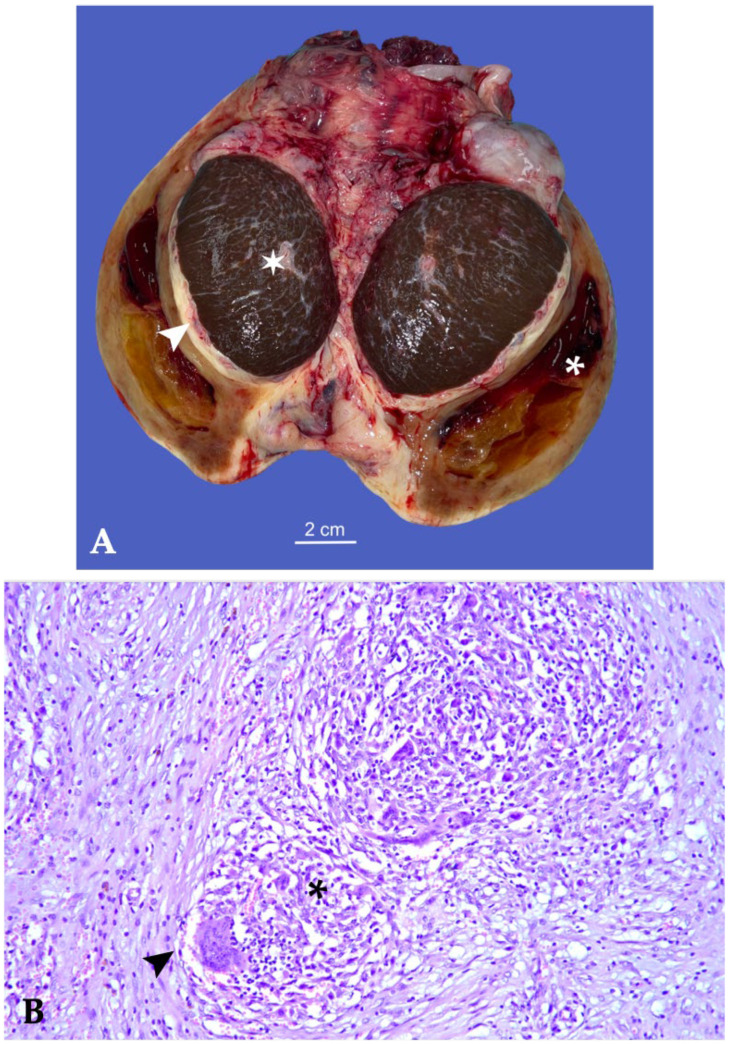
**Case 4.** Twelve-year-old Quarter Horse stallion with granulomatous periorchitis. (**A**) Gross appearance of the affected testis showing a markedly enlarged vaginal cavity containing abundant organized exudate with multiple septations and areas of coagulated material (asterisk). The tunica albuginea is markedly thickened (arrowhead), and the testicular parenchyma is brownish-red, atrophic, and diffusely affected by severe fibrotic changes (star). (**B**) Histological section of testis with granulomatous inflammation characterized by two coalescing granulomas composed of numerous epithelioid macrophages (asterisk), multinucleated giant cells (arrowhead), and non-degenerated neutrophils surrounded by inflamed fibrovascular stroma (H&E, 20×).

In Case 4, a 12-year-old Quarter Horse stallion, similar to Cases 2 and 3, four months after the initial drainage, repeat ultrasonographic examination revealed a “complex hydrocele” with multiple cystic compartments and heterogeneous echogenicity of the fluid. The testis showed an irregular echotexture with multifocal hyperechoic foci and diffuse thickening of the tunica albuginea. Because preservation of reproductive function was requested by the owner, unilateral orchiectomy was elected and performed under general anesthesia using a standard closed technique. Gross examination revealed a markedly enlarged right vaginal cavity measuring 16 × 9 × 7.5 cm, containing abundant organized exudate with multiple septations and areas of coagulated material. The tunica albuginea was diffusely thickened, measuring approximately 5 mm. The testicular parenchyma was brownish-red in color and atrophic, measuring approximately 8 × 6 cm, with increased firmness and extensive fibrotic changes (Figure 20A). In contrast to Cases 2 and 3, histopathological examination was available and demonstrated that the testicle was surrounded by a dense, thick, and organized inflammatory process composed of multiple granulomas on a rich fibrovascular stroma infiltrated by lymphocytes. The granulomas were composed of numerous elongate epithelioid macrophages and scattered multinucleated giant cells of foreign-type (Figure 20B). Special stains, such as Ziehl–Neelsen and Gram, for bacteria were negative. A secondary inflammatory reaction associated with the previous aspiration procedure was considered the most likely cause.

Histopathology of the two coalescing granulomas composed of numerous epithelioid macrophages, multinucleated giant cells, and non-degenerated neutrophils surrounded by inflamed fibrovascular stroma (H&E, 20×).

#### 3.3.2. Bacterial Epididymitis

A 6-year-old Martina Franca donkey was referred for evaluation of severe pain and swelling of the left testis.

Clinical examination revealed marked enlargement and tenderness of the left hemiscrotum, accompanied by discomfort with walking and fever. Pain was evident upon palpation of the scrotum and its contents, and the tail of the left epididymis was markedly enlarged. Scrotal enlargement, epididymal swelling, and pain on palpation were the predominant clinical findings. The right testis appeared normal in size and consistency.

Longitudinal ultrasonographic examination of the left epididymis revealed marked enlargement of the epididymis, particularly the tail, which was characterized by irregular margins, mixed echogenicity, and heterogeneous echotexture due to the presence of a large hypoechoic area consistent with abscess formation, later confirmed on gross examination.

A hypoechoic fluid accumulation containing internal echoes was observed between the layers of the tunica vaginalis, consistent with pyocele, which induced inflammatory thickening of the vaginal tunic. Periepididymal edema was also present, particularly around the head and body of the epididymis. Multifocal areas of mineralization were observed within the tunica vaginalis. These findings supported a presumptive diagnosis of septic epididymitis and orchitis complicated by pyocele. Ultrasonography played a key role in differentiating pyocele from hydrocele and in guiding the decision for prompt surgical intervention.

Bilateral surgical castration was performed under general anesthesia using a standard closed technique. Postoperatively, systemic antimicrobial and analgesic therapy were administered. Microbiological culture of the purulent material yielded *Streptococcus equi* subsp. *zooepidemicus*, and antimicrobial treatment was subsequently adjusted according to antibiogram results. Recovery was uneventful, with progressive and complete resolution of clinical signs. The donkey was monitored for four weeks postoperatively, during which no recurrence of scrotal swelling or postoperative complications was observed.

**Figure 21:**
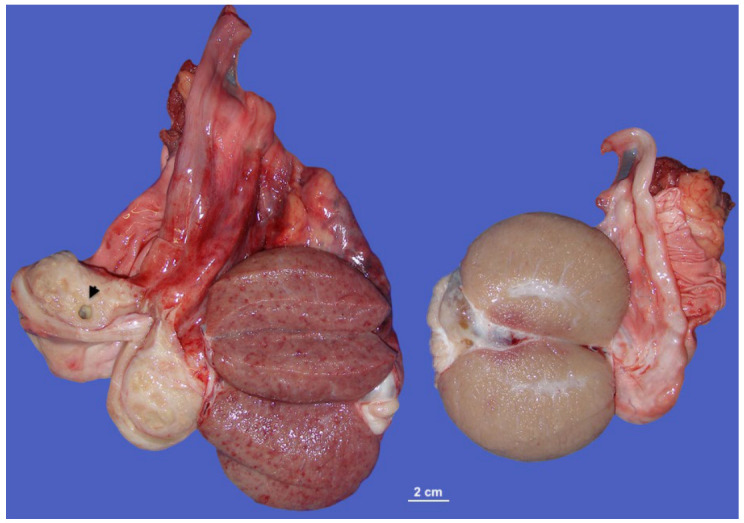
Six-year-old Martina Franca donkey with bacterial epididymitis caused by *Streptococcus equi* subsp. *zooepidemicus*. Gross appearance of the cut surface of the left testis and epididymis showing the gross lesions described above (**left**). The tail of the epididymis is markedly enlarged and contains an abscess (arrow). Controlateral testis (**right**).

On gross examination (Figure 21), the vaginal cavity showed diffuse reddish-brown discoloration, particularly involving the mesorchium and the spermatic vessels, consistent with severe inflammatory vascular injury. On cut surface, the left testis appeared reduced in size, measuring approximately 7 × 4 cm, with increased consistency and a reddish-brown coloration. The epididymis, particularly the tail, appeared markedly enlarged and firm due to the accumulation of suppurative inflammatory exudate. On cut section, a large cavitary lesion containing purulent material was present within the tail of the epididymis and extended into the surrounding tissues. Histopathological examination revealed chronic mild-to-moderate degeneration of the seminiferous epithelium in both testes. Additionally, the left testis showed mild multifocal lymphocytic interstitial orchitis associated with mildly degenerated seminiferous tubules, consistent with chronic inflammatory and degenerative changes secondary to infection. Examination of the tail of the epididymis revealed granulation tissue containing intermixed neutrophils, lymphocytes, and macrophages, supporting the diagnosis of chronic epididymitis.

The final diagnosis was consistent with bacterial epididymitis caused by *Streptococcus equi* subsp. *zooepidemicus*.

#### 3.3.3. Parasitic Orchitis

Four horses were diagnosed with parasitic inflammatory lesions involving the testes or associated genital structures (Table 4). Two horses were affected by *Strongylus vulgaris*, whereas two mixed-breed stallions presented lesions associated with *Setaria equina*. The two parasitic infections differed markedly in their anatomical localization and pathological features. *Strongylus vulgaris* was associated with intratesticular migratory lesions from the larva, whereas *Setaria equina* primarily involved the vaginal tunics, resulting in chronic eosinophilic granulomatous periorchitis.

Cases 1 and 2, two Quarter Horse stallions aged 3 and 4 years, respectively, were referred because of unilateral cryptorchidism. Transrectal ultrasonography confirmed a retained left abdominal testis in both horses.

**Figure 22:**
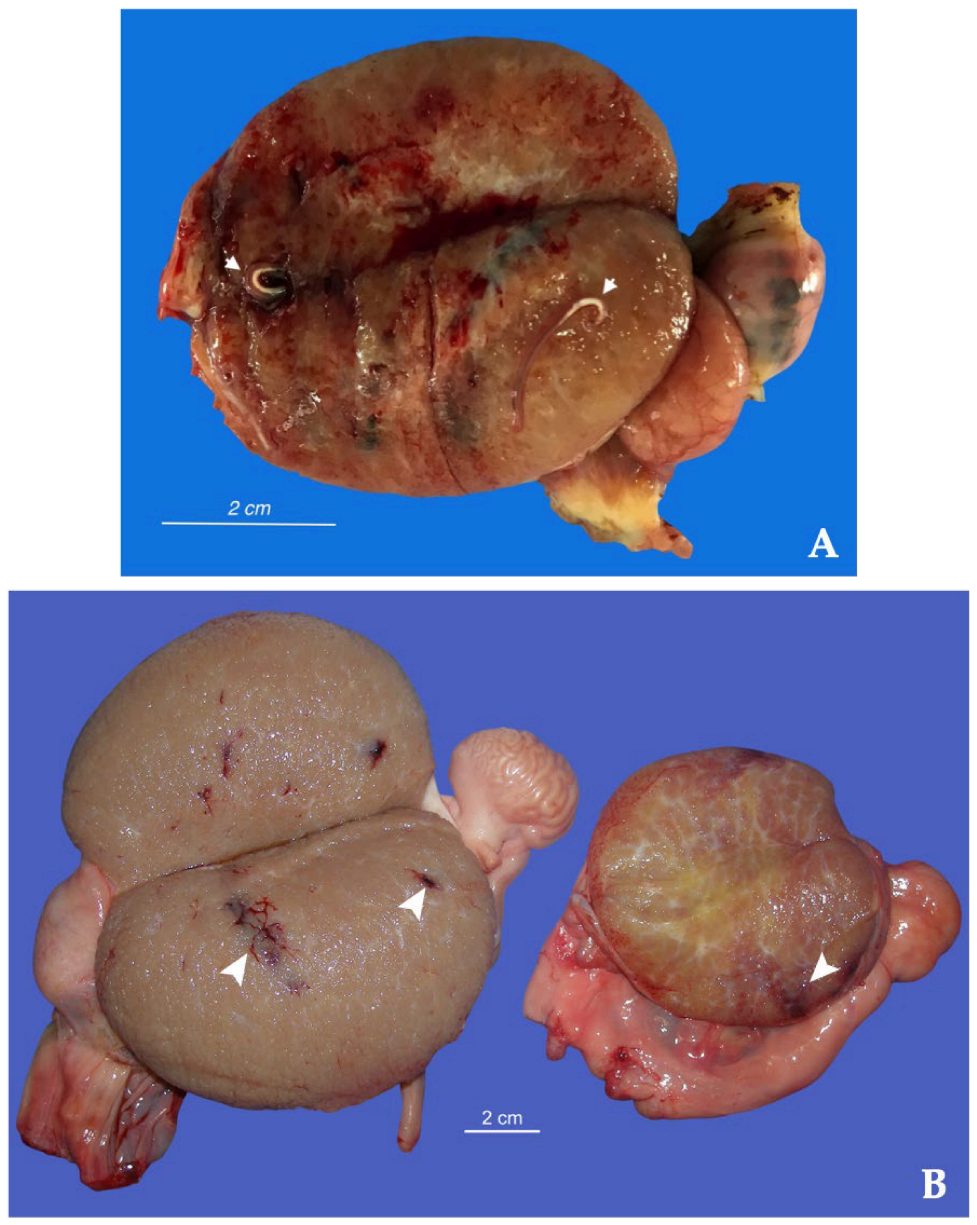
**Case 1.** (**A**) Three-year-old Quarter Horse stallion with *Strongylus vulgaris* orchitis. Gross appearance of the cut surface of the retained left cryptorchid testis showing two adult *Strongylus vulgaris* (arrows) emerging from hemorrhagic migratory tracts extending from the testicular parenchyma. (**B**) Case 2. Four-year-old Quarter Horse stallion with lesions consistent with previous *Strongylus* spp. migration. Gross appearance of the cut surfaces of the right scrotal testis (**left**) and the left retained cryptorchid testis (**right**) showing multiple hemorrhagic migratory tracts (arrowheads) extending from the peripheral toward the central testicular parenchyma. The lesions are particularly evident within the caudal pole and central region of the scrotal testis.

In case 1, ultrasonographic examination demonstrated a left abdominal cryptorchid testis measuring approximately 5 × 3 cm, and a heterogeneously hypoechogenic area was found surrounded by testicular tissue. In this area, a small 0.8-cm linear “train truck” structure, composed of two hyperechoic lines, was seen running parallel and resembling a cavitary cylindrical foreign body. A standing laparoscopic cryptorchidectomy was performed. Cut sections revealed the presence of two live adult worms coming out of 2-mm blood-filled tracks (Figure 22A) that extended from the tunica albuginea toward the center of the testis. Morphological identification confirmed one male and one female *Strongylus vulgaris.* Histopathological examination demonstrated chronic lymphoplasmacytic orchitis associated with mild fibrosis, abundant hemorrhage, and hypoplastic seminiferous tubules surrounding the parasitic migratory tracts. In addition, there was an abundant hemorrhage invading surrounding structures where hypoplastic seminiferous tubules were recognizable.

In case 2, gross examination (Figure 22B) of the left cryptorchid testis, which measured 7 × 4 cm, revealed multiple hemorrhagic tracts, approximately 2 mm in diameter, extending from the peripheral parenchyma toward the central portion of the testis. Similar lesions were also identified in the right scrotal testis, which measured 9.5 × 6 cm, where the hemorrhagic migratory tracts were particularly evident within the caudal pole and central region of the testicular parenchyma. No viable parasites were macroscopically identified within either testis. Histopathological examination of both testes revealed multifocal chronic hemorrhagic and inflammatory changes associated with intraparenchymal migratory tracts. These lesions were characterized by multifocal hemorrhage, mild fibrosis, and inflammatory infiltrates composed predominantly of lymphocytes, plasma cells, and scattered eosinophils. Based on the gross and histopathological findings, the lesions were considered highly suggestive of previous migration of *Strongylus* spp. larvae within the testicular parenchyma.

**Figure 23:**
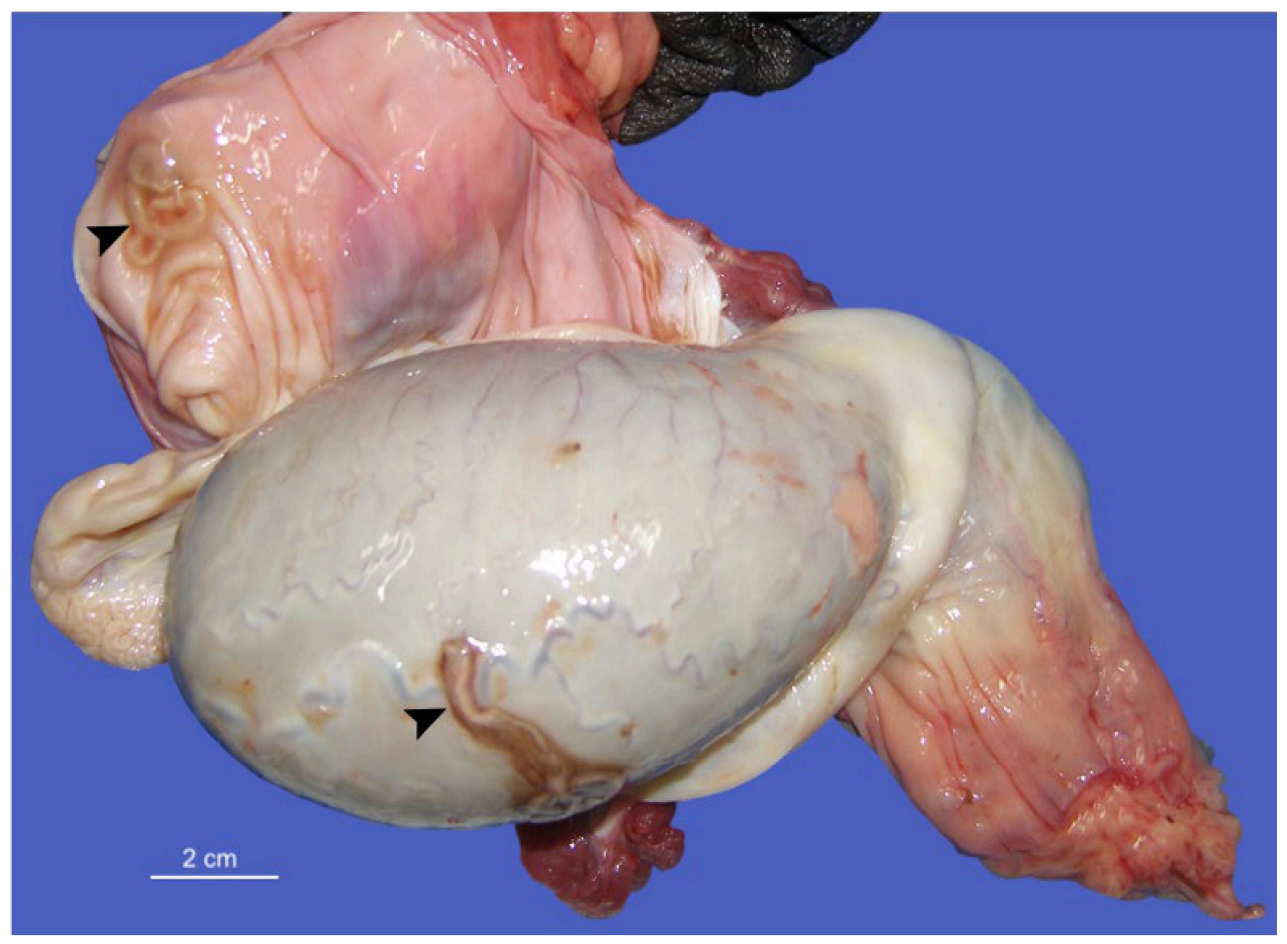
**Case 3.** Five-year-old mixed-breed stallion. Gross appearance of the two adult *Setaria equina* nematodes identified within the vaginal tunics (arrowheads). Note the marked thickening of the tunica albuginea associated with chronic eosinophilic granulomatous periorchitis.

Case 3 and 4, two mixed-breed stallions, aged 5 and 6 years, respectively, underwent elective orchiectomy at the owners’ request. Gross pathological examination revealed similar lesions in both cases, accompanied by nematodes. In the first animal, three free adult worms were identified within the vaginal cavity of the right testis. These findings were associated with periorchitis, characterized by elongated hemorrhagic streaks and marked edema of the epididymis. In the second animal, both testes were affected. Two adult nematodes were detected, one beneath the visceral tunica vaginalis and the other on the parietal tunica vaginalis of the right testis, surrounded by a localized reddish discoloration (Figure 23). A third parasite was found encapsulated within the parietal layer of the tunica vaginalis of the contralateral testis. In both subjects, inflammation of the appendix testis was evident bilaterally. Based on morphological criteria, the parasites were identified as *Setaria equina* in both cases. Upon incision of the vaginal cavity, a hydrocele was observed, containing a moderate volume of serous fluid ranging from yellow to pink. Both the parietal and visceral layers of the tunica vaginalis appeared thickened, with multiple fibrinous and fibrous adhesions. Acute lesions were characterized by extensive hemorrhagic infiltration involving adjacent tissues. Histopathological examination revealed severe multifocal to coalescing inflammation affecting the tunica vaginalis, characterized by hemorrhage, edema, and fibrovascular tissue proliferation. Frequent vasculitis and perivasculitis were observed and were associated with inflammatory infiltrates composed predominantly of lymphocytes, plasma cells, eosinophils, mast cells, and hemosiderin-laden macrophages. Multifocally, marked granulomatous inflammation composed of epithelioid macrophages, multinucleated giant cells, and numerous eosinophils was present. The testicular parenchyma retained active spermatogenesis in most areas; however, multifocal seminiferous tubular degeneration, peritubular edema, and mild focal lymphocytic orchitis were identified. Similar inflammatory changes extended into the epididymal tissues, where interstitial edema and multifocal inflammatory infiltrates were observed. Based on the gross, histopathological, and parasitological findings, a diagnosis of chronic eosinophilic granulomatous periorchitis associated with *Setaria equina* was established.

### 3.4. Neoplastic Disorders

Nine horses were diagnosed with testicular or scrotal neoplastic lesions (Table 5). Two of these cases have already been described in the previous sections because they were primarily classified according to other pathological conditions: Case 1, a Sertoli cell tumor arising in a horse with a disorder of sex development (Section 3.1), and Case 3, a Leydig (interstitial) cell tumor identified in a horse with congenital monorchidism (Section 3.2). The remaining seven cases are described below and include one malignant mixed sex cord–stromal tumor, one Leydig (interstitial) cell tumor arising in a cryptorchid testis, one Sertoli cell tumor associated with contralateral intratubular seminoma, three seminomas (including one metastatic malignant seminoma), and one scrotal sarcoid associated with secondary testicular degeneration.

#### 3.4.1. Malignant Mixed Sex Cord–Stromal Tumor

**Figure 24:**
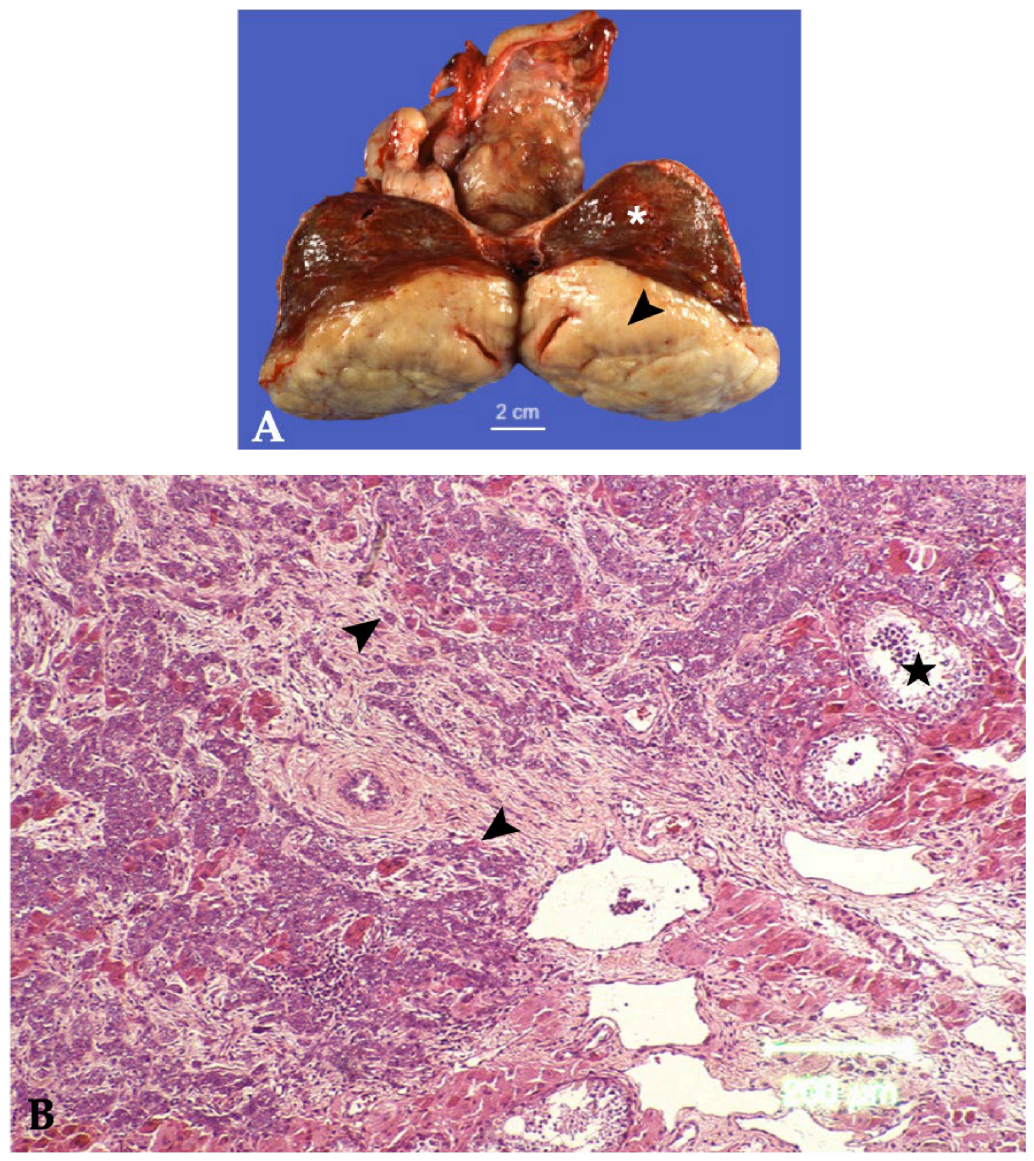
**Case 2.** Standardbred stallion, 30 years old. (**A**) Gross appearance of the cut surface of the gonad showing residual testicular parenchyma (asterisk) in the cranial portion and extensive replacement by a neoplastic mass (arrowhead). (**B**) Histological section of testis with malignant mixed sex cord–stromal tumor. Pleomorphic neoplastic Sertoli cells arranged in tubules (star) and cord (arrowhead) (H&E, 20×).

Case 2, a 30-year-old Standardbred stallion, was referred for a scrotal enlargement. The left testis was enlarged and firm; no heat or pain was detected on palpation. The right testis was normal in size. No involvement of the inguinal rings and iliac lymph nodes was evident on transrectal physical examination. Longitudinal and transverse ultrasonographic images showed a solid, nonhomogeneous tissue with thin hyperechoic bundles. As a testicular neoplasm was suspected, the stallion underwent standing unilateral orchiectomy to maintain reproductive function (owner’s request). Grossly, the testis measured 12 × 9 × 9 cm; was irregularly enlarged and firm; and showed evident superficial vascularization. On the cut surface, a solid neoplasm was observed, greyish-white in color, presenting a lobular pattern which extensively replaced the testicular parenchyma (Figure 24A). The latter, confined to the cranial extremity, was softer than normal, brown in color, and included four small neoplastic nodules (<6 mm). The epididymis was atrophic, and the head was not recognizable. At histological evaluation (Figure 24B), a dual Sertoli cell and Leydig cell tumor component was evident. Neoplastic Sertoli cells were prevalent, including some pleomorphic cells and frequent atypical mitotic figures. The tumor patterns displayed tubular, cord-like, and solid areas and occasional vascular invasion. The interstitial connective tissue was lowly fibrillar and contained neoplastic Leydig cells, with a lower grade of atypia. On the basis of these findings, a malignant sex cord–stromal tumor was diagnosed.

#### 3.4.2. Leydig (Interstitial) Cell Tumors

**Figure 25:**
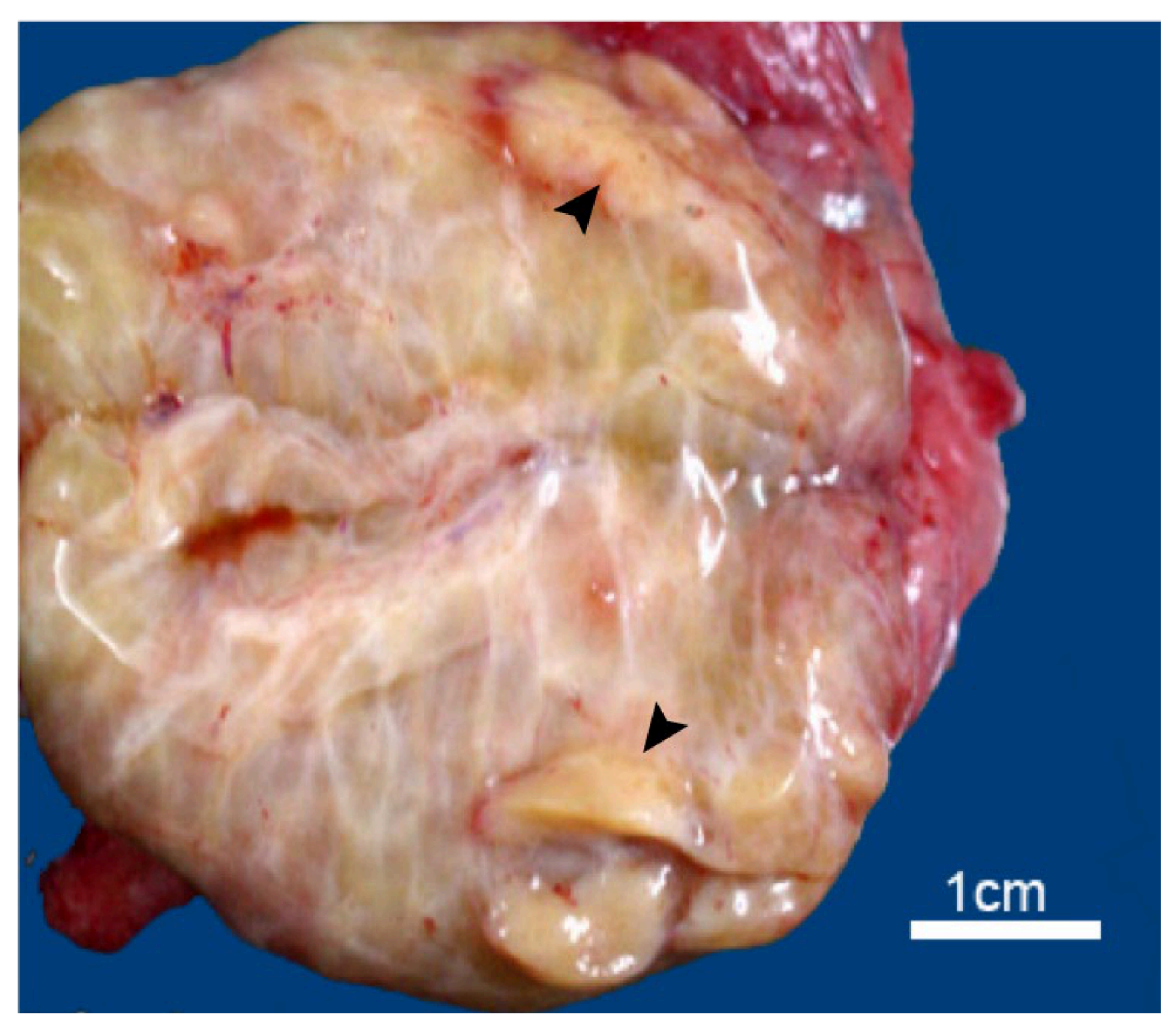
**Case 4.** Quarter Horse stallion, 5 years old. Gross appearance of the cut section of a cryptorchid testis with Leydig cell tumor showing yellowish-white neoplastic nodules (arrowheads).

Two horses were diagnosed with Leydig cell tumors. Case 3 occurred in a 7-year-old Appaloosa stallion with abdominal testis and monorchidism, whereas case 4 involved a 5-year-old Quarter Horse with left abdominal cryptorchidism. In case 4, ultrasonographic evaluation of the retained testis revealed multiple well-circumscribed, moderately hypoechoic nodules distributed throughout the testicular parenchyma, which measured approximately 1 cm in diameter. Gross examination (Figure 25) showed that the left cryptorchid testis measured 4.5 × 3 cm. On the cut surface, the parenchyma exhibited a gelatinous-like appearance. Round nodules, measuring 8–10 mm in diameter, were randomly distributed throughout the parenchyma and displayed the typical yellowish-white color. Histopathological examination of the cryptorchid testis revealed multiple expansile nodules of well-differentiated interstitial (Leydig) cells surrounding and entrapping immature seminiferous tubules. The neoplastic cells displayed abundant finely vacuolated cytoplasm and minimal cellular atypia, with rare mitotic figures.

#### 3.4.3. Sertoli Cell Tumor Associated with Intratubular Seminoma

**Figure 26:**
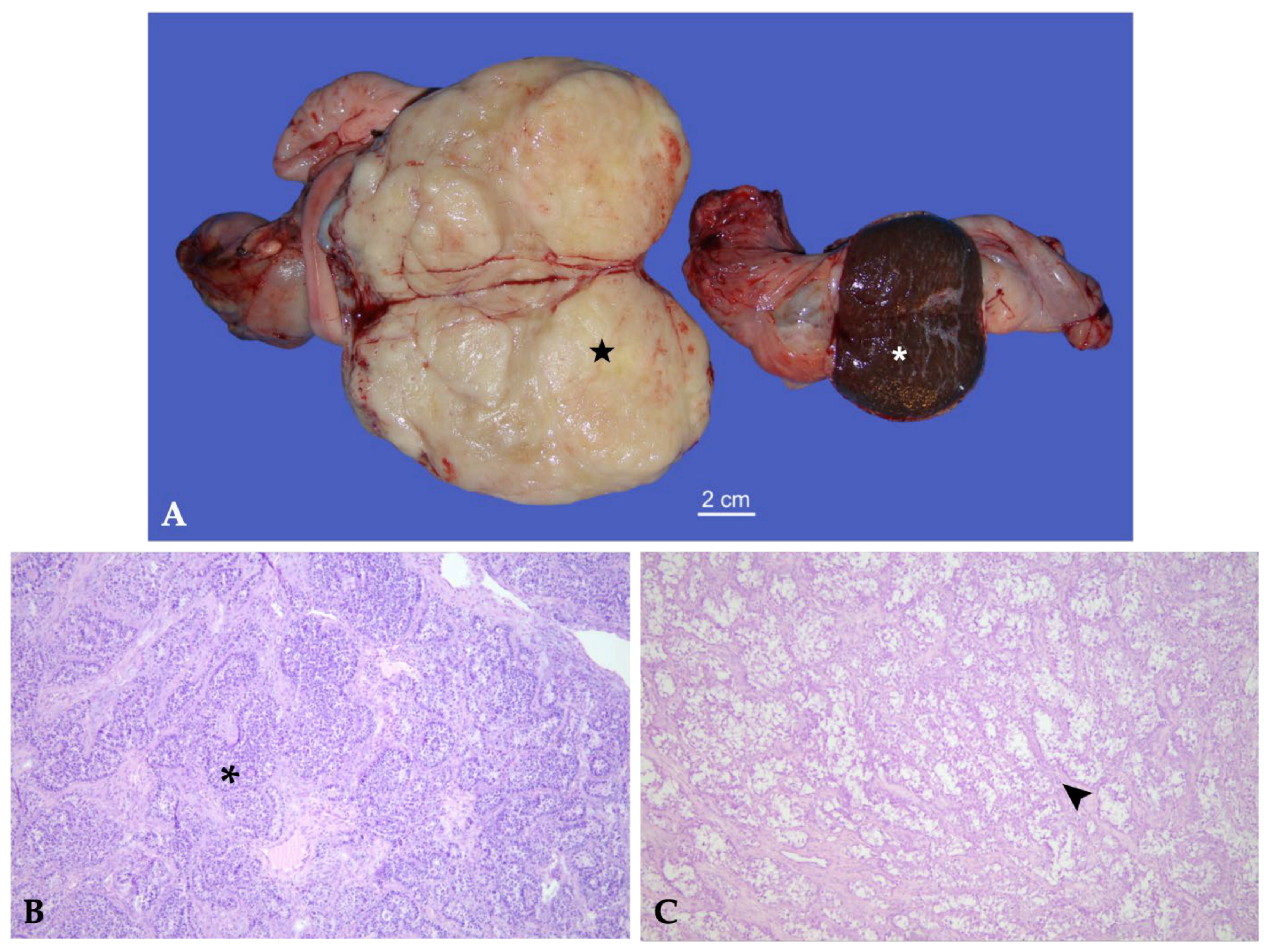
**Case 5.** Tolfetano stallion, 10 years old. (**A**) Gross appearance of the cut surface of both testes. The left testis is largely replaced by a well-circumscribed, encapsulated, whitish-grey mass infiltrating the testicular parenchyma while remaining confined within the tunica albuginea (star). The right testis appears reduced in volume, reddish-brown in color (asterisk). (**B**) Histological section of left testis with intratubular seminoma. Tubules are filled with neoplastic germ cells (asterisk) (H&E, 10×). (**C**) Histological section of right testis with Sertolioma. Numerous neoplastic tubules are lined by slender vacuolated Sertoli cells (arrowhead) (H&E, 10×).

Case 5 was a 10-year-old Tolfetano stallion presented with a history of enlargement of the right testis and reduction in size of the left testis. Ultrasonographic examination of the left testis revealed a large neoplastic lesion causing diffuse distortion of the normal testicular architecture. The residual testicular parenchyma surrounded the neoplastic mass, which was delineated by a thin hyperechoic rim. The neoplastic lesion exhibited a finely lobulated appearance and a heterogeneous echotexture characterized by alternating hypoechoic and hyperechoic areas, findings that were consistent with the gross pathological examination. Marked vascularization surrounding the mass and ectasia of the spermatic vessels were observed on Doppler ultrasonography. The right testis appeared markedly reduced in size and showed diffuse heterogeneous echotexture. The gross appearance of the left testis revealed a well-circumscribed and lobulated mass infiltrating the testicular parenchyma, with a whitish-grey coloration, measuring 14 × 8.5 cm. The tumor was completely contained within the tunica albuginea. The gross appearance of the right testis showed a reddish-brown color, reduced volume, and decreased consistency, measuring 4.5 × 3 cm (Figure 26A).

Histological evaluation diagnosed the presence of a diffuse Sertoli cell tumor in the right testis and an intratubular seminoma in the left testis (Figure 26B,C). In the right testis, a neoplasm composed of tubules, separated and surrounded by dense bands of fibrous connective tissue, was present. Neoplastic cells were columnar and had indistinct cell borders, a moderate amount of granular cytoplasm with frequent clear vacuoles, and a round to oval nucleus with granular chromatin and distinct nucleoli. Cells frequently palisaded along the basement membrane of tubules. In the left testis, the neoplasm was composed of round cells with distinct cell borders that filled the lumens of seminiferous tubules, replacing spermatogenic and Sertoli cells. Neoplastic cells had a scant cytoplasm and large, round, vesicular nuclei with granular chromatin and prominent nucleoli. Anisocytosis and anisokaryosis were moderate. Mitotic figures were few.

#### 3.4.4. Seminomas

**Figure 27:**
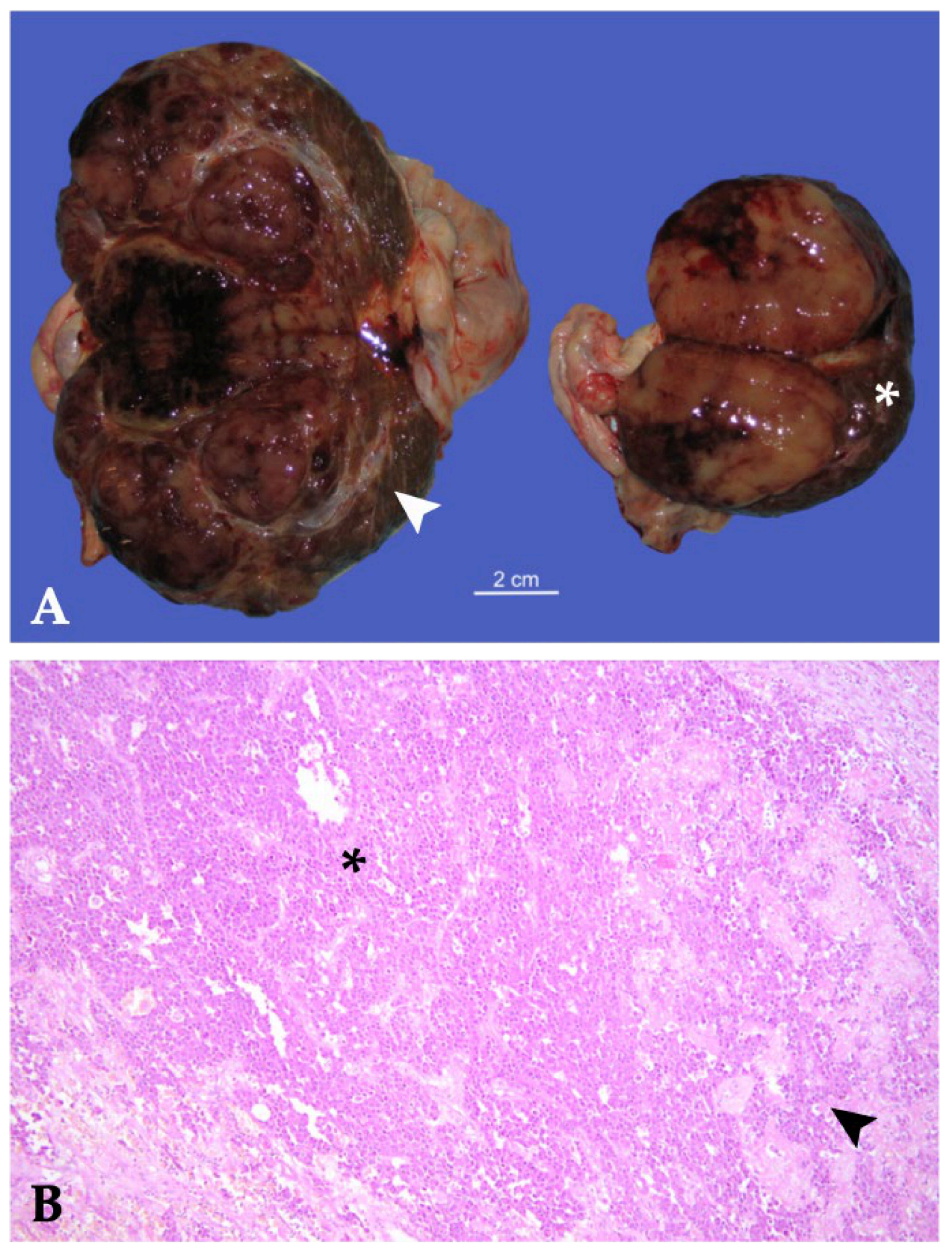
**Case 6.** Paint Horse stallion, 17 years old. (**A**) Gross appearance of the cut surfaces of both testes affected by bilateral diffuse seminoma. The right testis is extensively replaced by a grayish-pink, lobulated neoplastic proliferation composed of multiple coalescing nodules, with only a small remnant of residual testicular tissue visible near the cranial pole (asterisk). The left testis shows replacement of the parenchyma by a well-demarcated, yellowish neoplastic mass protruding from the cut surface, with a small residual area of testicular tissue remaining at the caudal pole (arrow head). (**B**) Histological section of testis with diffuse seminoma: sheets (asterisk) and cords (arrow head) of round cells with scant cytoplasm replacing tubules arranged in a diffuse pattern (H&E, 10×).

Three horses (Cases 6–8) were diagnosed with seminoma, including one case of bilateral diffuse seminoma, one unilateral diffuse seminoma, and one metastatic malignant seminoma. Case 6 was a 17-year-old American Paint Horse stallion. On detailed examination of the scrotal and testicular structures, the right testis appeared markedly enlarged, with a smooth surface, uniformly firm consistency, and was non-painful upon palpation. The epididymis showed increased consistency and a pronounced separation from the superior margin of the testis. The spermatic cord appeared enlarged in volume, but without changes in consistency. The left testis was normal in size but had increased consistency, as did the epididymis. Ultrasonographic examination of the right testis revealed a heterogeneous parenchyma characterized by macro- and micro-lobulation, with near-total replacement of the normal testicular tissue. At the level of the spermatic cord, ectasia of the vascular structures was observed. In contrast, the echotexture of the left testis showed a well-defined area measuring approximately 7 × 5 cm, less echogenic and with a coarser texture compared to the residual testicular parenchyma, which was detectable only near the caudal pole. The tail of the epididymis in both testes appeared uniformly echogenic. Rectal palpation did not reveal any abnormalities of the iliac lymph nodes. The absence of clinical signs indicative of an inflammatory condition, supported by the anamnesis and, above all, by the ultrasonographic patterns, allowed for the formulation of a diagnosis of bilateral testicular neoplasia. The animal subsequently underwent orchiectomy in a standing position. On gross pathological examination (Figure 27A), the right testis measured 11 × 10 × 8 cm, with a rounded shape, smooth surface, and firm consistency. On cut section, the normal parenchyma was replaced by a proliferative, lobulated newformed tissue, with nodules ranging from 2–3 mm to 3–4 cm in diameter, and grayish-pink coloration. A small area of residual testicular tissue was present near the cranial pole, containing small grayish-pink nodular formations. Gross examination revealed grayish areas apparently outlining the neoplastic nodules toward the cranial pole. In addition, a stellate brown area, suggestive of necrosis, was observed near the caudal pole. These features emphasize the heterogeneous gross appearance of the seminoma. The epididymis appeared reduced in size. The left testis measured 9 × 6.5 × 5 cm, with a smooth surface and firm consistency. On section, there was clear replacement of the parenchyma by a well-defined, protruding newformed tissue of slightly increased consistency, mildly lobulated, and yellowish in color. A small residual area of testicular parenchyma was observed at the caudal pole. Histologically (Figure 27B), a neoplastic process developing within the tubules—filling them and replacing spermatogenic and Sertoli cells—that also largely extended into the interstitium, as cords and sheets in a solid and diffuse pattern. This process consisted of a proliferation of round cells (germ cells) with generally distinct cell borders and scant to moderate granular eosinophilic cytoplasm; the nuclei were large, round, and vesicular, containing finely stippled or coarsely clumped chromatin and one or two very prominent nucleoli. The N:C ratio was high, anisocytosis and anisokaryosis were moderate, and mitotic activity ranged from 1 to 4 mitoses per HPF. These intratubular neoplastic foci showed focal necrosis.

Case 7, a 10-year-old saddle horse stallion, was referred for evaluation of an enlarged left testis. On palpation, the affected testis was enlarged, non-painful, and showed variable consistency. Ultrasonographic examination revealed a heterogeneous appearance of the left testis, with a consistently multilobulated neoplastic lesion.

The testicular parenchyma appeared diffusely hypoechoic, with poorly defined hyperechoic regions corresponding to a pseudo-capsule, creating a clear demarcation line between the nodular neoplastic lesion and a small residual peripheral portion of normal testicular parenchyma. The right testis appeared normal, with homogeneous echogenicity and preserved architecture of the testicular parenchyma.

The stallion was admitted for surgical treatment under general anesthesia, and bilateral castration was performed using a standard closed technique.

**Figure 28:**
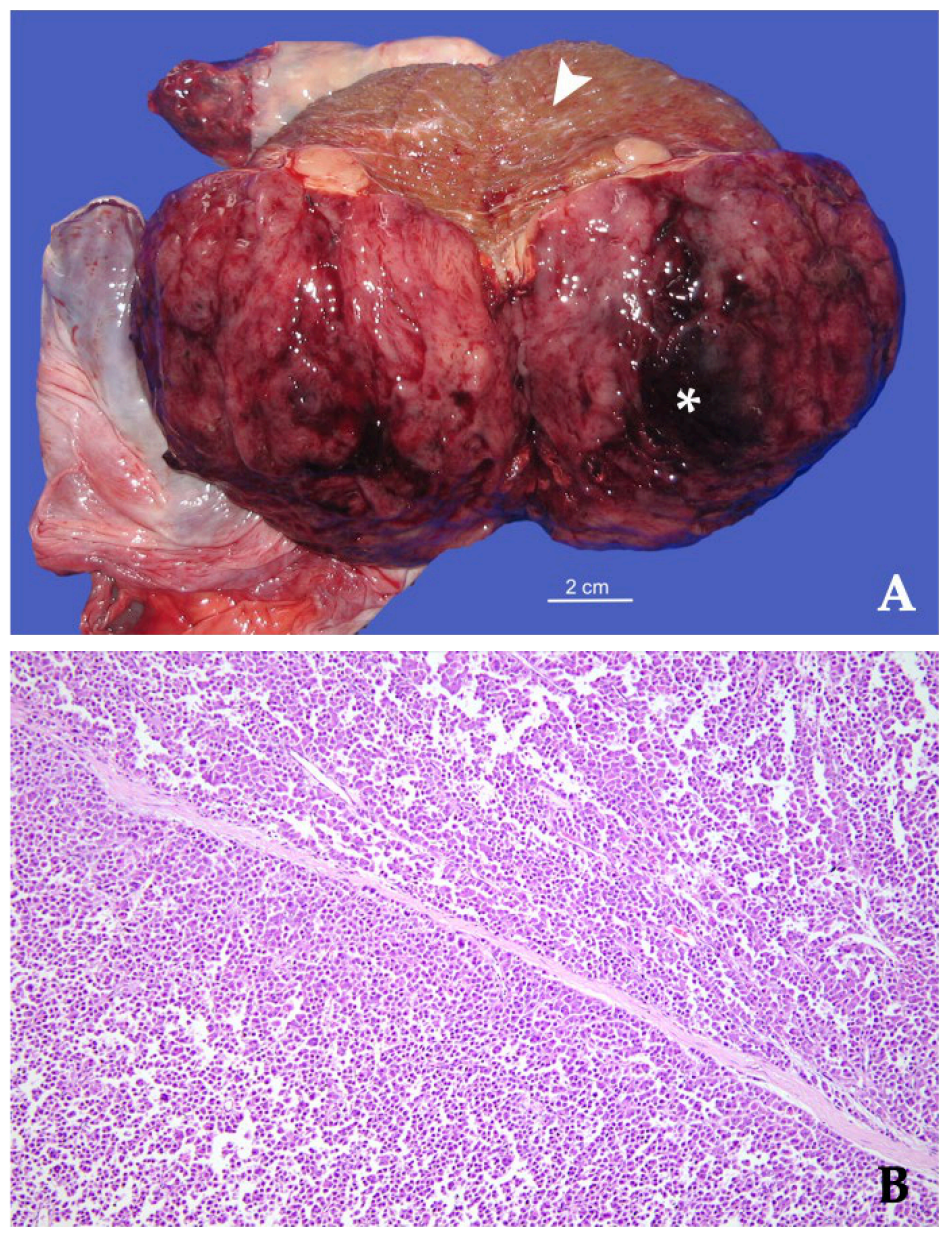
**Case 7.** Saddle Horse stallion, 10 years old. (**A**) Gross appearance of the testis on cut section. The parenchyma is almost entirely replaced by a grayish-pink neoplastic mass, with only a thin peripheral layer of residual testicular tissue remaining (arrow head). Multifocal bright-red areas within the lesion indicate prominent neoangiogenesis (asterisk). (**B**) Histological section of testis with diffuse seminoma: sheets of round cells with scant cytoplasm replacing tubules arranged in a diffuse pattern (H&E, 10×).

At gross examination (Figure 28A), the left testis measured 11 × 10 cm, whereas the neoplastic nodule measured 8 × 8.5 cm. The mass bulged from the testicular parenchyma, was surrounded by a thin capsule, and almost completely replaced the normal testicular parenchyma. On the cut section, the central portion of the mass showed viable tissue with neoangiogenesis, evidenced by bright red areas. The neoplastic nodule was grayish-pink in color. Histopathological examination of the testis revealed a diffuse seminoma (Figure 28B) characterized by a neoplastic proliferation of germ cells developing within and expanding the seminiferous tubules, where the neoplastic population replaced both spermatogenic and Sertoli cells. The neoplasm also extended into the interstitial tissue, forming diffuse solid sheets. Neoplastic cells were round, with generally distinct cell borders and scant to moderate eosinophilic granular cytoplasm. Nuclei were large, round, and vesicular, containing finely stippled to coarsely aggregated chromatin and one to two prominent nucleoli. The nucleus-to-cytoplasm ratio was high, while anisocytosis and anisokaryosis were moderate. Mitotic activity ranged from 1 to 4 mitotic figures per high-power field. Multifocally, intratubular neoplastic foci contained scattered necrotic cells. These findings were consistent with a diffuse seminoma.

**Figure 29:**
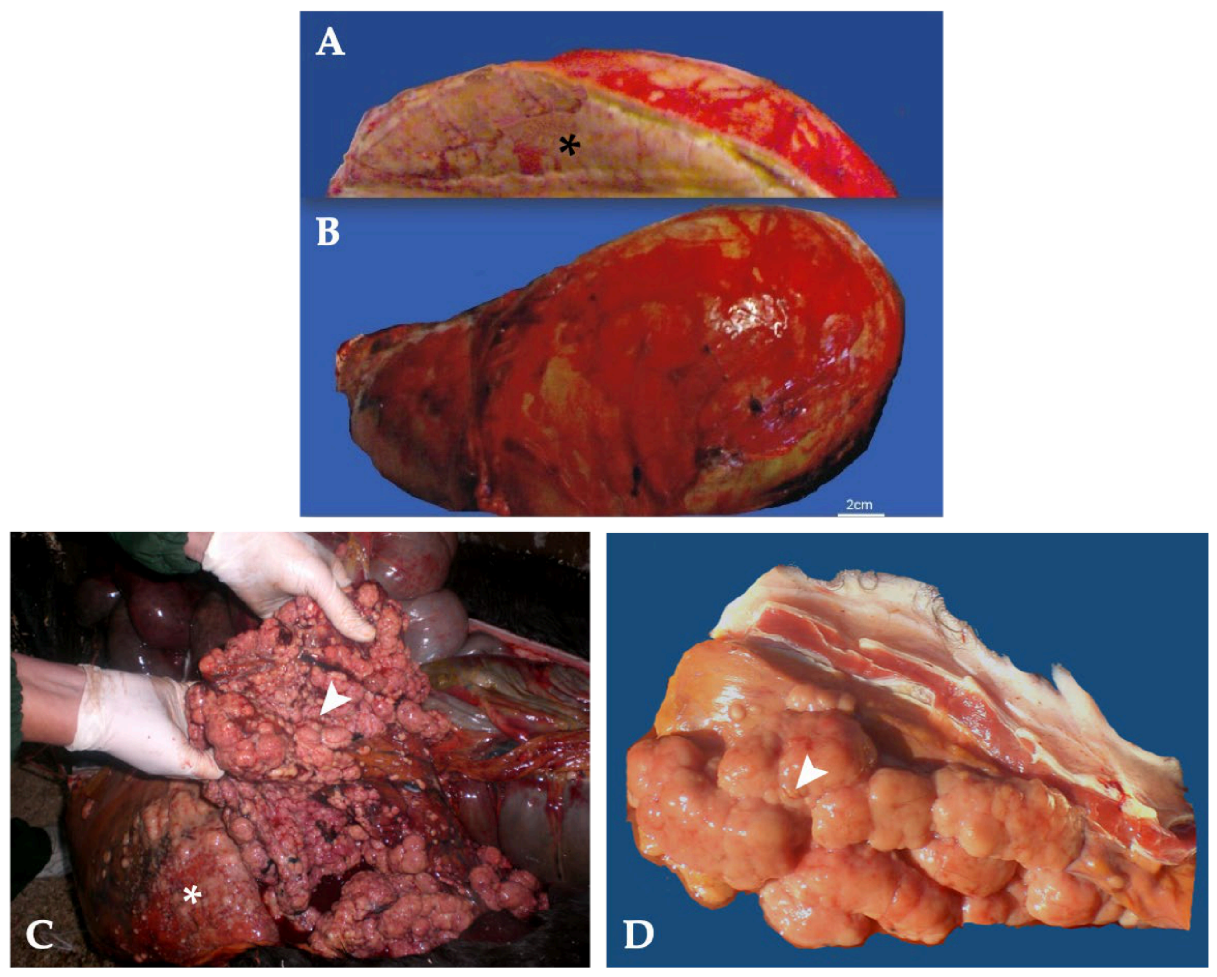
**Case 8.** Saddle Horse stallion, 27 years old. (**B**) Gross appearance of the enlarged left testis affected by seminoma. (**A**) Cut surface showing extensive replacement of the testicular parenchyma by a pale tan neoplastic mass (asterisk). (**C**) Multiple metastatic lesions of the malignant seminoma involving the lung (asterisk), liver (arrowhead), and mesentery. (**D**) Diffuse metastatic implants distributed over the parietal peritoneum (arrowhead).

Case 8, a 27-year-old Saddle Horse stallion, was referred by a private practitioner for pathological evaluation following unilateral orchiectomy performed because of marked enlargement of the left hemiscrotum. According to the clinical history provided by the referring veterinarian, the left testis was markedly enlarged, firm, and non-painful on palpation, measuring approximately three times the size of the contralateral testis, which appeared reduced in size. The affected left testis measured 24.5 × 12.5 cm. According to the information provided by the referring practitioner, ultrasonographic examination revealed severe disruption of the normal testicular architecture, with replacement of the parenchyma by a heterogeneous multilobulated mass. Assessment of the iliac lymph nodes was not performed during the initial diagnostic work-up. In retrospect, the presence of metastatic involvement of the iliac lymph nodes might have suggested disseminated disease before surgery and could have influenced the diagnostic assessment and therapeutic decision-making. The stallion subsequently underwent unilateral orchiectomy under general anesthesia using a standard closed technique. Gross examination (Figure 29A,B) revealed a pale tan, bulging, multilobulated mass, with a gray-white surface, extensively replacing the normal testicular parenchyma on cut section. According to the follow-up information provided by the referring practitioner, four months after surgery, the stallion was readmitted because of anorexia and diffuse swelling of the left hind limb. Owing to the poor prognosis and progressive clinical deterioration, the owner elected euthanasia. Necropsy examination revealed multiple tan, semi-firm plaques and pedunculated nodules diffusely distributed over the parietal and visceral peritoneum, liver and lung, consistent with extensive metastatic dissemination (Figure 29C,D). Based on the histopathological and necropsy findings, a final diagnosis of metastatic malignant seminoma was established.

#### 3.4.5. Scrotal Sarcoid Associated with Secondary Testicular Degeneration

**Figure 30:**
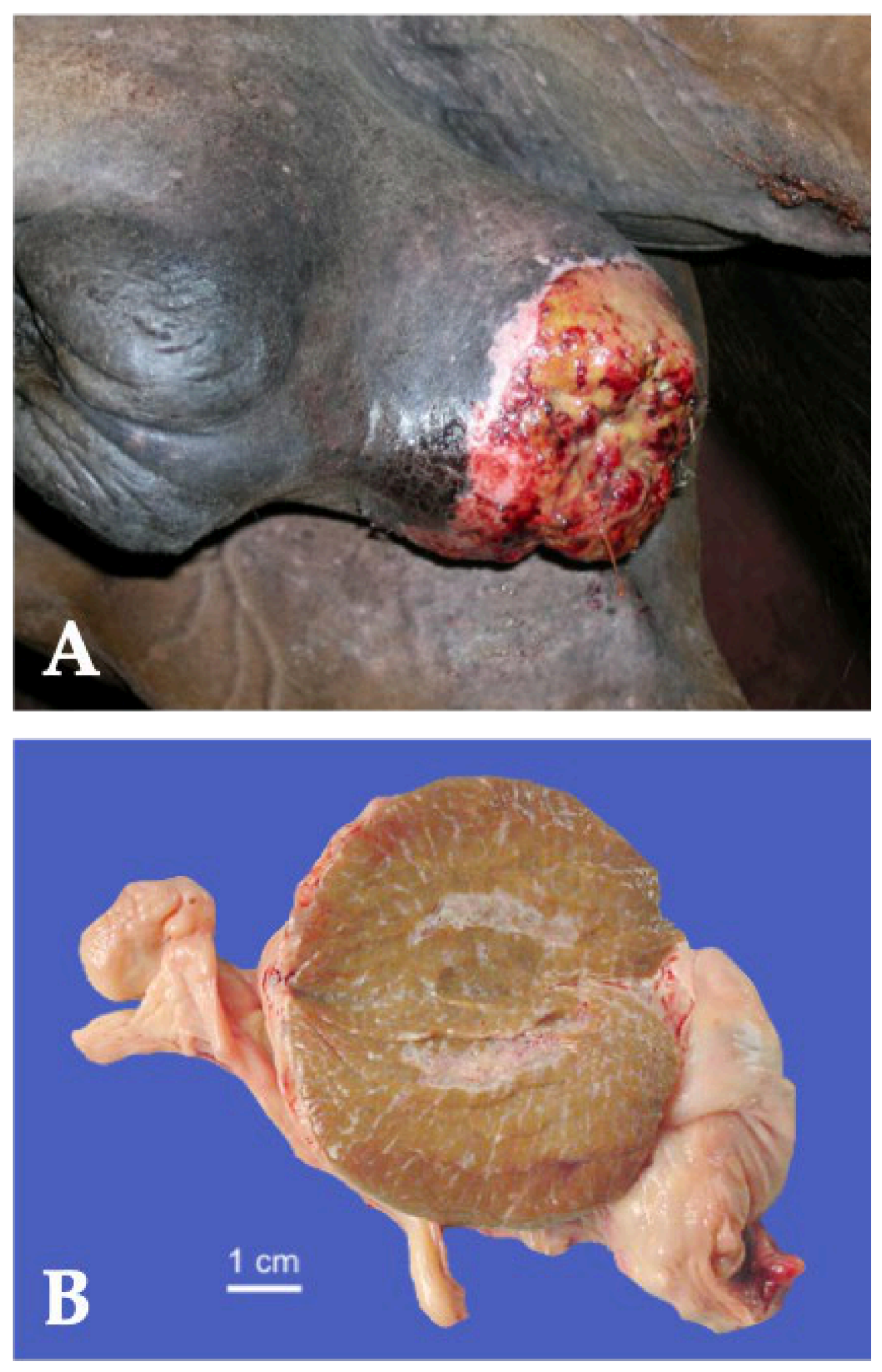
**Case 9.** Thoroughbred (PSI) stallion, 9 years old. (**A**) Gross appearance of a wart-like lesion measuring approximately 5 × 6 cm located on the left hemiscrotum. (**B**) Testicular parenchyma compressed by the adjacent sarcoid, characterized by marked degeneration, loss of normal architecture, and absence of the typical protruding appearance on cut section.

A 9-year-old PSI horse was referred for evaluation of a mass located on the scrotum. A wart-like lesion measuring approximately 5 × 6 cm was identified on the left hemiscrotum (Figure 30A). The solid mass projected into the scrotal cavity, causing a visible depression on the testicular parenchyma. Surgical excision of the mass was performed with ablation of the scrotum, and an elective bilateral orchiectomy was also carried out using a standard closed technique. The diagnosis of sarcoid was confirmed by histological evaluation. The left testis, compressed by the sarcoid, resulted in degeneration (Figure 30B). Histopathological examination confirmed the diagnosis of sarcoid, characterized by a non-encapsulated proliferation of spindle-shaped fibroblastic cells arranged in interlacing bundles beneath a hyperplastic epidermis. The adjacent left testis exhibited diffuse degenerative changes characterized by seminiferous tubular atrophy, reduced spermatogenesis, thickening of tubular basement membranes, and mild interstitial fibrosis. Multifocal degeneration and loss of germ cells were present, while the remaining seminiferous tubules showed variable degrees of spermatogenic depletion. These findings were consistent with secondary chronic compression-induced testicular degeneration.

## 4. Discussion

This retrospective study evaluated 57 equine testicular disorders diagnosed over a 26-year period (Table 6). The cases encompassed a broad range of congenital, developmental, inflammatory, degenerative, traumatic, and neoplastic conditions, highlighting the remarkable diversity of equine testicular pathology and diagnostic challenges associated with these disorders.

Disorders of sexual development (DSDs) represented an important subset of congenital abnormalities and demonstrated considerable phenotypic and cytogenetic variability. Clinical presentation ranged from isolated cryptorchidism to severe malformations of the external genitalia, while cytogenetic analyses revealed normal XY horses, sex chromosome mosaicism, and SRY-negative XX individuals. These findings emphasize the complexity of equine DSDs and support the use of a multidisciplinary diagnostic approach that combines clinical examination, imaging, surgery, histopathology, and when available, cytogenetic testing [30,31,32,33,34,35,36,37].

Other uncommon congenital abnormalities identified in this series included monorchidism, cystic ectasia of the rete testis, and ectopic adrenal cortical tissue, all of which are rarely reported in equids. Only a limited number of equine monorchidism have been reported previously [38,39,40,41,42,43,44]. In our series, laparoscopy proved invaluable for confirming monorchidism and differentiating true testicular agenesis from complete prenatal degeneration of the retained testis, reinforcing its role as diagnostic gold standard.

Congenital cystic ectasia of the rete testis was identified in one abdominal cryptorchid testis, consistent with the few reports describing this lesion in retained equine testes [45,46,47]. Comparable lesions have been described in humans, an alpaca, and an Angus bull, although in those species the affected testes were scrotal rather than retained [48,49]. In the present case, ultrasonography accurately identified the lesion before surgery, while histopathology confirmed the diagnosis.

Ectopic adrenal cortical tissue within the testicular tissue is seldom reported in horses. Previous studies have documented its presence at the base of the spermatic cord, between the epididymal head and the testis, and within the mediastinum testis [6,16]. Other locations include the kidney, retroperitoneal tissue, broad ligament, ovaries, and testes [6,15,16,17]. The absence of associated pathological changes in the present case suggests that this lesion represents an incidental developmental finding without apparent clinical significance.

Acquired disorders were dominated by testicular degeneration, spermatic cord torsion, inflammatory disease, and neoplasia. Testicular degeneration occurred secondary to severe underlying conditions, including failed laparoscopic castration without orchiectomy, anabolic androgenic steroid administration [50,51,52], chronic hydrocele, and prolonged compression. These findings emphasize that testicular degeneration should be regarded as the final common pathological response to a variety of insults rather than a primary disease process. In particular, persistence of functional testicular tissue following laparoscopic spermatic cord transection without orchiectomy supports previous recommendations [53] that this technique should not be considered an acceptable alternative to complete castration.

Spermatic cord torsion represented an uncommon but clinically important surgical emergency. Although the typical presentation consisted of acute scrotal pain and enlargement, the identification of clinically silent torsion in a retained abdominal testis illustrates the variability of this condition [21,54,55]. Doppler ultrasonography consistently identified severe vascular compromise and proved to be an essential tool for diagnosis and surgical decision-making [2,13,19,20,21,55,56,57,58].

Inflammatory lesions included hydrocele, parasitic orchitis, chronic periorchitis, and bacterial epididymitis with orchitis. Hydrocele was associated with progressive chronic inflammatory and degenerative changes. Although often considered a benign condition, persistent hydrocele may lead to progressive testicular degeneration secondary to chronic compression and may be associated with fibrosis of the vaginal tunics and chronic periorchitis [6,54]. Bilateral orchiectomy represented an appropriate treatment considering the chronicity of the lesions and the irreversible structural changes affecting both the vaginal tunics and the testicular parenchyma. Repeated aspiration was consistently followed by the development of a “complex” hydrocele characterized by fibrous septation, chronic periorchitis, and irreversible testicular degeneration, suggesting centesis should not be considered definitive treatment.

*Strongylus vulgaris*, *Strongylus edentatus*, and *Setaria equina* are the parasites most frequently associated with orchitis, epididymitis, and periorchitis in horses [14,16,59,60]. The present series further expands the pathological spectrum of these lesions. Although individual cases of *Strongylus vulgaris* infection in a cryptorchid testis [14] and *Setaria equina* with associated periorchitis [59] have previously been reported by our group, their inclusion in this larger retrospective series highlights the diversity of parasitic lesions affecting both retained and scrotal testes. These findings emphasize that parasitic infection should be included among the differential diagnoses of inflammatory testicular disease, particularly in cryptorchid horses.

Bacterial infection represented another uncommon inflammatory cause of testicular pathology. Although bacterial orchitis and epididymitis have occasionally been reported in horses, to the authors’ knowledge, no detailed descriptions are currently available in donkeys [22,23,24]. For this reason, this case was included because it represents a particularly unusual and clinically relevant presentation. The donkey presented with severe unilateral scrotal enlargement, marked epididymal swelling, pain, and pyrexia, consistent with previous descriptions of bacterial epididymitis with orchitis in horses [22,24,61,62]. Bilateral orchiectomy combined with culture-guided antimicrobial therapy resulted in complete clinical recovery, confirming that prompt surgical treatment remains the treatment of choice for severe bacterial orchitis and epididymitis [22].

Testicular neoplasm represented a diverse and uncommon group of lesions in horses, most commonly unilateral [16]. Seminoma represented the most frequent neoplasm in our series, followed by Leydig cell tumors, mixed sex cord–stromal tumors, and Sertoli cell tumors, reflecting the distribution generally reported in the literature [12,16]. Although ultrasonographic findings were not specific for individual tumor types, they consistently identified distortion of the normal testicular architecture and facilitated surgical planning. Histopathological examination remained essential for definitive diagnosis, tumor classification, and assessment of the biological behavior [26,63]. Nevertheless, testicular tumors should always be differentiated from other causes of testicular or scrotal enlargement, including orchitis, epididymitis, hydrocele, varicocele, hematoma, scrotal hernia, and spermatic cord torsion [54].

Seminoma was the most frequently encountered neoplasm in the present series, consistent with previous reports identifying it as the most common testicular tumor in mature stallions [1,12,16,25,54,64,65,66]. Although most equine seminomas are unilateral [67], bilateral involvement has occasionally been described [63,68] and was also identified in one horse of the present study. Of particular interest, one horse developed a testicular seminoma that demonstrated metastatic potential and was associated with poor long-term prognosis. This finding is consistent with published case series describing metastasis of testicular seminoma to locations such as abdominal organs and the thoracic cavity [63,65,66,67,69].

Leydig cell tumors represented the second most frequent neoplasm in this series and occurred exclusively in abdominal cryptorchid testes, supporting previous reports [70,71,72].

Sertoli cell tumors remain uncommon in horses and have been described in both scrotal and retained testes, occasionally demonstrating malignant behavior [14,73,74,75,76]. The most remarkable case in the present series involved a Sertoli cell tumor arising in the subcutaneous testis of a 3-year-old Arabian horse with a disorder of sexual development and a 64, XX, SRY-negative karyotype. The coexistence of a DSD, bilateral gonadal hypoplasia, cryptorchidism, and Sertoli cell neoplasia is exceptionally uncommon in horses and further emphasizes the importance of complete pathological and cytogenetic evaluation in animals presenting with disorders of sexual development.

Mixed sex cord–stromal tumors are exceptionally rare in horses and are characterized by the coexistence of Sertoli and Leydig cell components within the same neoplasm [77,78]. The malignant mixed sex cord–stromal tumor included in the present series has previously been described by our group [79] and is reported here to complete the pathological spectrum identified during the study period.

One of the principal findings of this study was the consistent diagnostic value of ultrasonography across all disease categories. Although sonographic findings were rarely pathognomonic, ultrasonography reliably differentiated congenital, inflammatory, degenerative, and neoplastic conditions; guided surgical planning; and identified lesions requiring histopathological confirmation. Similarly, histopathological examination frequently revealed clinically unsuspected lesions and proved indispensable for establishing definitive diagnoses, particularly in congenital abnormalities and neoplastic conditions. These findings support routine submission of excised testes for pathological evaluation whenever possible.

This study has limitations inherent to its retrospective design, including variability in medical records, diagnostic investigations, and treatment protocols over the 26-year study period. Advances in imaging technology and pathological interpretation may also have influenced case evaluation. Nevertheless, the large number of cases and the comprehensive integration of clinical, ultrasonographic, surgical, gross pathological, and histopathological findings provide a valuable reference for veterinarians managing equine testicular disorders.

## 5. Conclusions

In conclusion, equine testicular diseases comprise a diverse group of congenital and acquired disorders that often present with similar clinical signs despite markedly different pathological processes. Accurate diagnosis relies on integrating physical examination, ultrasonography, surgical exploration, and histopathological evaluation. Routine pathological examination of excised testes should be encouraged because clinically unsuspected lesions may otherwise remain undetected. Collectively, this case series provides a comprehensive clinicopathological reference that may facilitate the diagnosis, differential diagnosis, treatment, and clinical management of stallions with testicular disease.

## Figures and Tables

**Table 1:** Clinical, cytogenetic and pathological findings in the six horses diagnosed with disorders of sexual development (DSDs) (PCR: Polymerase Chain Reaction).

Case	Breed	Age	Main Clinical Findings	Testicular Location	Cytogenetics	Main Pathological Diagnosis
1	Arabian	5 years	Underdevelopment of the prepuce and penis, absence of the scrotum, no libido	Bilateral abdominal	64, XY, SRY-positive (PCR)	Bilateral severe testicular hypoplasia
2	Italian Saddle Horse	15 months	Abnormal external genitalia, micropenis, absence of the scrotum, enlarged inguinal teats, stallion-like behavior	Bilateral subcutaneous	64, XX/64, XY/63, XO, SRY-positive (PCR)	Bilateral severe testicular hypoplasia
3	Arabian	3 years	Abnormal external genitalia, micropenis, absence of the scrotum, enlarged inguinal teats, stallion-like behavior	Left abdominal, right subcutaneous	64, XX, SRY-negative (PCR)	Bilateral severe hypoplasia with unilateral Sertoli cell tumor
4	Arabian	18 months	Abnormal external genitalia, micropenis, absence of the scrotum, enlarged inguinal teats, stallion-like behavior	Bilateral subcutaneous	Not performed	Bilateral severe testicular hypoplasia
5	Trotter	2 years	Abnormal external genitalia, absence of the scrotum, micropenis associated with epispadias, stallion-like behavior	Bilateral subcutaneous	Not performed	Bilateral severe testicular hypoplasia
6	Andalusian	3 years	Bilateral cryptorchidism	Bilateral abdominal	64, XY, SRY-positive (PCR)	Rudimentary uterus replacing the ductus deferens

**Table 2:** Clinical characteristics of horses diagnosed with spermatic cord torsion.

Case	Breed	Age (Years)	Affected Testis	Degree of Torsion	Main Clinical Signs
1	Quarter Horse	6	Left abdominal (cryptorchid)	>360°	No clinical signs; incidental intraoperative finding during bilateral cryptorchidectomy
2	Missouri Fox Trotter	12	Right scrotal	270°	Acute colic, marked hemiscrotal enlargement, enlarged firm painful testis
3	Trotter	5	Left scrotal	>420°	Acute severe scrotal pain, marked hemiscrotal enlargement, mild hydrocele, cranial displacement of the epididymal tail

**Table 3:** Clinical characteristics of horses with hydrocele and periorchitis.

Case	Breed	Age (Years)	Clinical Presentation	Ultrasonographic Findings	Gross Findings	Histopathology	Outcome
1	Italian Saddle Horse	11	Right hemiscrotal enlargement	Hydrocele	Diffuse thickening of the tunica vaginalis	Not available	Bilateral orchiectomy
2	Andalusian	5	Recurrent left hemiscrotal enlargement following hydrocele aspiration	Complex hydrocele	Organized serofibrinous exudate	Not available	Bilateral orchiectomy
3	Friesian	7	Recurrent left hemiscrotal enlargement following hydrocele aspiration	Complex hydrocele	Organized serofibrinous exudate	Not available	Bilateral orchiectomy
4	Quarter Horse	12	Recurrent left hemiscrotal enlargement following hydrocele aspiration	Complex hydrocele	Organized exudate and fibrosis	Granulomatous periorchitis	Unilateral orchiectomy

**Table 4:** Clinical and pathological characteristics of horses with parasitic orchitis and periorchitis.

Case	Breed	Age (Years)	Parasite	Parasite Localization	Main Lesions
1	Quarter Horse	3	*Strongylus vulgaris*	Testicular parenchyma (left retained testis)	Hemorrhagic migratory tracts, chronic orchitis
2	Quarter Horse	4	*Strongylus* spp. (suspected)	Testicular parenchyma (left retained and right scrotal testis)	Hemorrhagic migratory tracts, chronic orchitis
3	Mixed breed	5	*Setaria equina*	Vaginal cavity and tunica vaginalis (scrotal testis)	Hydrocele, eosinophilic granulomatous periorchitis
4	Mixed breed	6	*Setaria equina*	Vaginal cavity and tunica vaginalis (bilateral scrotal testis)	Hydrocele, eosinophilic granulomatous periorchitis

**Table 5:** Clinical, ultrasonographic and pathological findings in horses diagnosed with neoplastic lesions.

Case	Breed	Age	Main Clinical Findings	Affected Testis	Ultrasonographic Findings	Final Diagnosis
1	Arabian	3 years	DSDs	Right subcutaneous	Not reported	Sertoli cell tumor associated with bilateral severe testicular hypoplasia (64, XX SRY-negative)
2	Standardbred	30 years	Progressive unilateral scrotal enlargement	Left scrotal	Large heterogeneous solid mass with thin hyperechoic bundles	Malignant mixed sex cord–stromal tumor
3	Appaloosa	7 years	Monorchidism	Left abdominal	Heterogeneous abdominal testis with fibrous connective tissue network	Leydig (interstitial) cell tumor
4	Quarter Horse	5 years	Left abdominal cryptorchidism	Left abdominal	Multiple well-circumscribed hypoechoic intratesticular nodules	Leydig (interstitial) cell tumor
5	Tolfetano	10 years	Left testicular enlargement with contralateral testicular atrophy	Bilateral scrotal	Lobulated heterogeneous left testicular mass with marked vascularization	Sertoli cell tumor associated with intratubular seminoma
6	American Paint Horse	17 years	Progressive enlargement of the right testis	Bilateral scrotal	Bilateral heterogeneous multilobulated masses replacing normal parenchyma	Bilateral diffuse seminoma
7	Saddle Horse	10 years	Left testicular enlargement	Left scrotal	Multilobulated heterogeneous intratesticular mass with pseudocapsule	Diffuse seminoma
8	Saddle Horse	27 years	Marked unilateral testicular enlargement	Left scrotal	Heterogeneous multilobulated mass with complete disruption of the normal architecture	Metastatic malignant seminoma
9	Thoroughbred	9 years	Scrotal mass causing compression of the testis	Left hemiscrotum	Not reported	Scrotal sarcoid associated with secondary testicular degeneration

**Table 6:** Percentages were calculated using the total number of equids included in the study (*n* = 57) with testicular disorders.

Testicular Disorder	N. of Cases	Percentage (%)
Disorders of Sexual Development (DSDs)	5	8.77
Disorders of Sexual Development (DSDs) with Sertoli Cell Tumor	1	1.75
Monorchidism	1	1.75
Monorchidism with Leydig (Interstitial) Cell Tumor	1	1.75
Cystic Ectasia of the Rete Testis	1	1.75
Testicular Adrenal Rest Tissue	1	1.75
Ischemic Necrosis Following Failed Laparoscopic Castration	2	3.51
Bilateral Testicular Degeneration Associated with Suspected Anabolic Steroid Administration	1	1.75
Spermatic Cord Torsion	3	5.26
Compensatory Hypertrophy and Hyperplasia in Retained Testes	24	42.11
Intratesticular Cyst	1	1.75
Hydrocele	1	1.75
Periorchitis	2	3.51
Bacterial Epididymitis	1	1.75
Parasitic Orchitis/Periorchitis *Strongylus vulgaris*	2	3.51
Parasitic Orchitis/Periorchitis *Setaria equina*	2	3.51
Malignant Mixed Sex Cord–Stromal Tumor	1	1.75
Leydig (Interstitial) Cell Tumor	1	1.75
Sertoli Cell Tumor	1	1.75
Seminoma	4	7.02
Scrotal Sarcoid Associated with Secondary Testicular Degeneration	1	1.75

## Data Availability

The data presented in this study are available from the first author upon reasonable request.
